# Regulation of multi-area power system load frequency in presence of V2G scheme

**DOI:** 10.1371/journal.pone.0291463

**Published:** 2023-09-11

**Authors:** Mahmoud M. Hussein, Tarek Hassan Mohamed, Mohamed Metwally Mahmoud, Mansour Aljohania, Mohamed I. Mosaad, Ammar M. Hassan

**Affiliations:** 1 Department of Electrical Engineering, Faculty of Energy Engineering, Aswan University, Aswan, Egypt; 2 Electrical & Electronics Engineering Technology Department, Yanbu Industrial College (YIC), Royal Commission Yanbu Colleges & Institutes, Yanbu, Saudi Arabia; 3 Electrical Engineering Department, Faculty of Engineering, Damietta University, Damietta, Egypt; 4 Arab Academy for Science, Technology and Maritime Transport, South Valley Branch, Aswan, Egypt; J.C. Bose University of Science and Technology, YMCA, INDIA, INDIA

## Abstract

The integration of renewable sources (RSs) and the widespread deployment of electric vehicles (EVs) has transitioned from a luxury to a necessity in modern power systems. This results from the sharp increase in electric power demand and public awareness of switching to green energy. However, in addition to load fluctuations and changes in system parameters, these RSs and EVs negatively impact the load frequency (LF). This work presents a LF control for a modern multi-area power system incorporating photovoltaic (PV) and EV chargers. The proposed controller primarily utilizes EV chargers within modern power systems. This approach offers the advantage of using the already present components instead of introducing new ones. The proposed controller comprises the ecological optimization approach (ECO) and the integral controller (I). Both of these components are designed for autonomous vehicle-to-grid (V2G) devices. The proposed control technique is applied to a three-area power system, where the V2G scheme is located in Area-1. Variations in the load, PV power generated, and system parameters are considered to evaluate the effectiveness of the proposed (I+ECO+V2G) controller for controlling the LF. To assess the performance of the proposed I+ECO+V2G system, a comparative analysis is conducted to compare its performance with both the I+ECO system and the standard I-controller. The simulation findings demonstrate that implementing the I+ECO and the proposed I+ECO+V2G strategies results in enhanced system stability and decreased LF fluctuations compared to the conventional I-control approach. Furthermore, while comparing the I+ECO control technique to the suggested control strategy I+ECO+V2G, it was seen that the latter reaches steady state values more quickly. The results validate the robustness and effectiveness of the proposed controller in mitigating the impacts of load disturbances, uncertainties, and nonlinearities within the system. These simulations were performed using MATLAB/SIMULINK. To validate the outcomes of the simulation results, an experimental setup consisting of a real-time dSPACE DS1103 connected to another PC via QUARC pid_e data acquisition card was used. The experimental findings have substantiated the accuracy of the simulation findings about the superiority of the I+ECO+V2G methodology compared to both the I+ECO and I-control methodologies concerning system performance and LF control.

## 1. Introduction

### a) Background

In recent years, renewable energy sources (RESs) are already a part of the new electric networks. Most countries started to rely entirely on them and are moving toward doing away with fossil fuels. Photovoltaic (PV) and wind powers are the two most often used RESs by, 228 GW and 906 GW, respectively [1–3]. In addition to RESs, electric vehicles (EVs) have started to be employed as a viable substitute for those that rely on fossil fuels in order to maintain green energy growth and energy efficiency [4, 5]. One of the primary concerns associated with using Renewable Energy Sources (RESs) is the adverse impact on the load-frequency-control (LFC) of the power system (PS). This is due to the reliance of the energy produced by RESs on environmental changes, such as temperature and solar radiation for the PV systems and the wind speed for wind systems [6, 7]. While for EVs, the frequency is affected by power electronic devices (inverter–rectifier) that connect them into the grid [8].

Achieving a balance between power output and customer demand is paramount for properly functioning a power system. This entails effectively managing fluctuations in both the load and supply side. A PS’s-LFC helps tie-line-connected multi-area PSs operate steadily in the steady state. Power exchanges across linked areas, the unpredictability of distributed generators, model hypotheses, and power requirements all contribute to the complexity of the LFC problem [9, 10]. Automatic voltage regulators (AVR) have been used to reduce active-reactive power losses while maintaining a consistent standard voltage. Variations in operational voltage have a significant impact on the resilience of the PS. Due to the severe non-linear and volatility of PSs and the growing adoption of RESs, integrated AVR-LFC techniques are crucial. As a result, conventional AVR-LFC methods were placed to the test [11, 12]. Current EVs and RES incorporation add significant complexity to the PS, which puts its ability to function under intense pressure. Some advanced control strategy is required to prevent a scenario like this. To optimize the performance of the PSs, it is essential to implement a control approach that is sufficiently effective to mitigate any potential vulnerabilities [11, 13, 14]. Based on the aforementioned information, the research studies relevant to the current field and their primary shortcomings are reviewed in the subsequent part.

### b) Literature review

Numerous studies on PS regulation have been conducted in recent years. For the LFC of PSs, various cutting-edge control approaches have been created, such as distributed control, optimal control, resilient control, fuzzy control, predictive control, and mixed control algorithms [15, 16]. A cooperative control-based technique for small power plants frequency and voltage (F&V)control was introduced and is based on V control. For F-V control, a non-linear threshold-based control technique was suggested [17]. The PS control based on machine learning was demonstrated in [15]. Concurrent F-V control was examined using a state estimation-based controller. A thorough analysis of the PS active and reactive power control coupling features was provided [12]. The influence of imbalanced grid voltage on active and reactive powers was investigated experimentally, and the coupling characteristics between F&V for PSs were also examined [18]. For F&V robustness under system unpredictability, coordinated control of PSs was investigated. In addition to these, other gain-tuning optimization-based controllers are offered. To enhance hybrid PS performance, a more sophisticated cascaded control method is investigated. The PS is based on a sliding surface predictive controller [17].

The vehicle-to-grid (V2G) scheme is considered one of the most important techniques for controlling the PS LFC. In this scheme, EVs have been exploited during the periods of the EVs plug-in. This can be done according to a pre-scheduled agreement with the EVs owners to not interfere with their interests. In [18, 19], the concept of V2G has been utilized using the EVs batteries in order to inject power into the grid. By discharging their excess EV storage energy into the utility grid when the price of electricity is high, the authors have used the V2G scheme to reduce the cost of charging by about 13.6%. In addition, EVs attracted attention as a pollution-free power storage system for periods of urgent need of power. However, authors in [20, 21], have indicated that the EVs usage is increased in case of isolated grids. The authors in [21, 22], have presented some publications that exploit EVs or V2G arrangements in PSs and discussed their assistance in controlling the PS–LFC. For two-area (thermal and hydrothermal) PSs with the aid of EVs, a unique optimised cascading fuzzy-fractional order integral derivative with a filter regulator was used to quickly alleviate fluctuations in the ’PS’ frequency [23]. On EV integration for reconstructed PSs, significant effort was completed. Various RESs were used to explore the use of EVs in deregulated LFC processes. Authors have assessed the benefits of employing EVs in V2G schemes for PSs as well as the overall operation of EVs. A management plan for household systems with adjustable loads and EVs linked with RESs has been presented by authors in based on the benefits of EVs. The study involved a combined F-V control using EVs. It was completed to LFC a hybrid PS with RESs and V2G system [9, 12, 23].

The use of current control approaches significantly aids the ability to modulate of PSs. It is essential to maintain system control under emergencies and rapid load variants, like when there is the prospect of temporary disruption, in order to avoid any aberrant scenarios in the system. In order to address this issue, many controllers for PS assessment of quality have been created and implemented in the existing literature. With its various configurations, the PID controller is the simplest and easiest frequency control mechanism available for interconnected PSs. The PID control may be implemented for F-control [24–26]. As interconnected PSs integrated with RESs like PV and EV are non-linear systems with high nonlinearities, some metaheuristic optimization techniques should be employed for fine-tuning the controller parameters. Among these techniques are Jaya, slap swarm, social-spider optimizer, and wild horse [27–30]. Despite the optimized PID controller’s performance in most applications in achieving the control aim, they suffer from tuning the gain values at the rated operating conditions. Therefore, changing this operating point could not result in the best performance, which is, seen as a weakness of this controller.

Many adaptive control techniques were employed to overcome the fixed optimized gains of the PID controller. These techniques rely on changing the controller parameter, which in turn affects the control signal, in response to changes in the operating point or system characteristics. Model reference adaptive PI controller was presented for PV systems [31]. Model-free adaptive and model reference adaptive controllers were studied in wind energy systems [32, 33]. In addition, artificial neural networks and adaptive neuro-fuzzy inference systems for load frequency control were investigated [24]. On the other hand, some attempts have been made to use the ecological sign stability algorithm as an optimal controller in PSs [34]. According to the ecological theory, in case of parameter variations, improvement of the system robustness is related to the sign stability of the nominal parameters matrix. Ecology and biology population fields deal with the nature of the population in terms of increase or decrease and the conflict between them for domination over others. In [35, 36], the authors have proposed many population mathematical models. The authors in [37], have presented a coefficient diagram method with ecological technique as a decentralized controller for adjusting the LFC in the interconnected PS. The results via this method are very satisfactory.

### c) Contributions

This work presented an Integral controller and ECO during EVs plug-in, V2G. This controller (I-ECO-V2G) is presented for the LFC of an interconnected PS. This system is a three-area PS that is incorporated with PVs.

The main contributions of this work can be summarized as:

- The effect of plugging on/off some EV units on the system frequency is elaborated.
- Implementing I-ECO-V2G control technique for LFC in a multiple-area PS that contains PV.
- Investigate the performance of the suggested controller with variations in the amount of PV power generated and certain load fluctuations.
- Study the impact of the suggested controller for F-regulation under system parameters changes, including turbine and governor’s time constants.
- Compare the performance of the suggested controller (I + ECO + V2G) with other controllers (I and I + ECO) for LFC under certain variations (variations in the amount of PV power generated, certain load fluctuations, and system parameters changes, including turbine and governor’s time constants).
- MATLAB/SIMULINK and real-time simulation (RTS) results demonstrate the high performance of the proposed controller on the examined system.

### d) Paper organisation

This manuscript is organized as the following; Section (2) presents the dynamic model of the studied system. Section (3) designates the smart grid with EVs, including the V2G system. The ecological technique is indicated in Section (4). Section (5) presents the execution scheme of the (I + ECO +V2G) in the PS containing three-area. Section (6) gives the output results and discussions. The conclusions are drawn in Section (7).

## 2. System dynamic model

**Fig 1:**
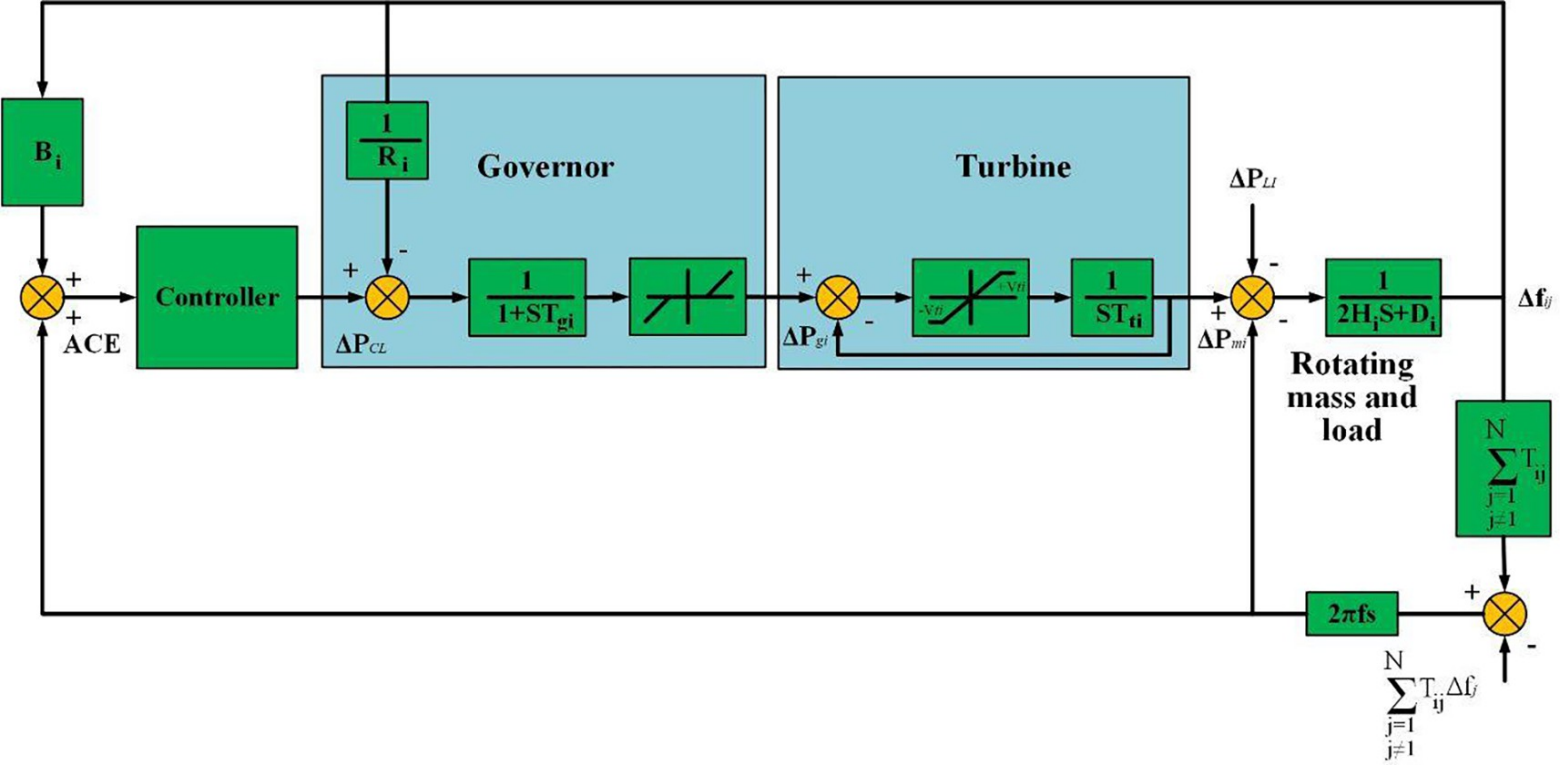
Configuration of the interconnected power system.

This section of the manuscript describes the model of a multi-area LFC-PS considering the tie-line power signal. Fig 1 illustrates a generalized PS with N-control areas. A turbine produces mechanical power for the generator unit, which converts it into electrical power [35, 36]. Due to the difficulties of storing large amounts of electrical power, a balance between the total power generated and the total load demand should be achieved. The PS dynamic model could be described in the subsequent state space model as indicated in the following equations [36].

$$[\begin{array}{c} \Delta {\dot{P}}_{gi}\\ \Delta {\dot{P}}_{mi}\\ \Delta {\dot{f}}_{i}\\ \Delta {\dot{P}}_{tie,i} \end{array}]=[\begin{array}{cccc} -\frac{1}{T_{S}} & 0 & -\frac{1}{R_{i}T_{gi}} & 0\\ \frac{1}{T_{ti}} & -\frac{1}{T_{ti}} & 0 & 0\\ 0 & \frac{1}{2H_{i}} & -\frac{D}{2H_{i}} & \frac{1}{2H_{i}}\\ 0 & 0 & 2\pi \sum_{j=1,j\neq i}^{N}\;T_{ij} & 0 \end{array}][\begin{array}{c} \Delta P_{gi}\\ \Delta P_{mi}\\ \Delta f_{i}\\ \Delta P_{tie,i} \end{array}]+[\begin{array}{ll} 0 & 0\\ 0 & 0\\ -\frac{1}{2H_{i}} & 0\\ 0 & 2\pi \end{array}][\begin{array}{l} \Delta P_{Li}\\ \Delta P_{vi} \end{array}]+[\begin{array}{c} \frac{1}{T_{S}}\\ 0\\ 0\\ 0 \end{array}]\Delta P_{d}$$ (1)

$$y_{i}={ACE}_{i}=[0\;0\;B_{i}\;1]\;\left[\begin{array}{c} \Delta P_{gi}\\ \Delta P_{mi}\\ \Delta f_{i}\\ \Delta P_{tie,i} \end{array}\right]$$ (2)

where (.) represents a differential operator.

## 3. Smart grid with EVs

**Fig 2:**
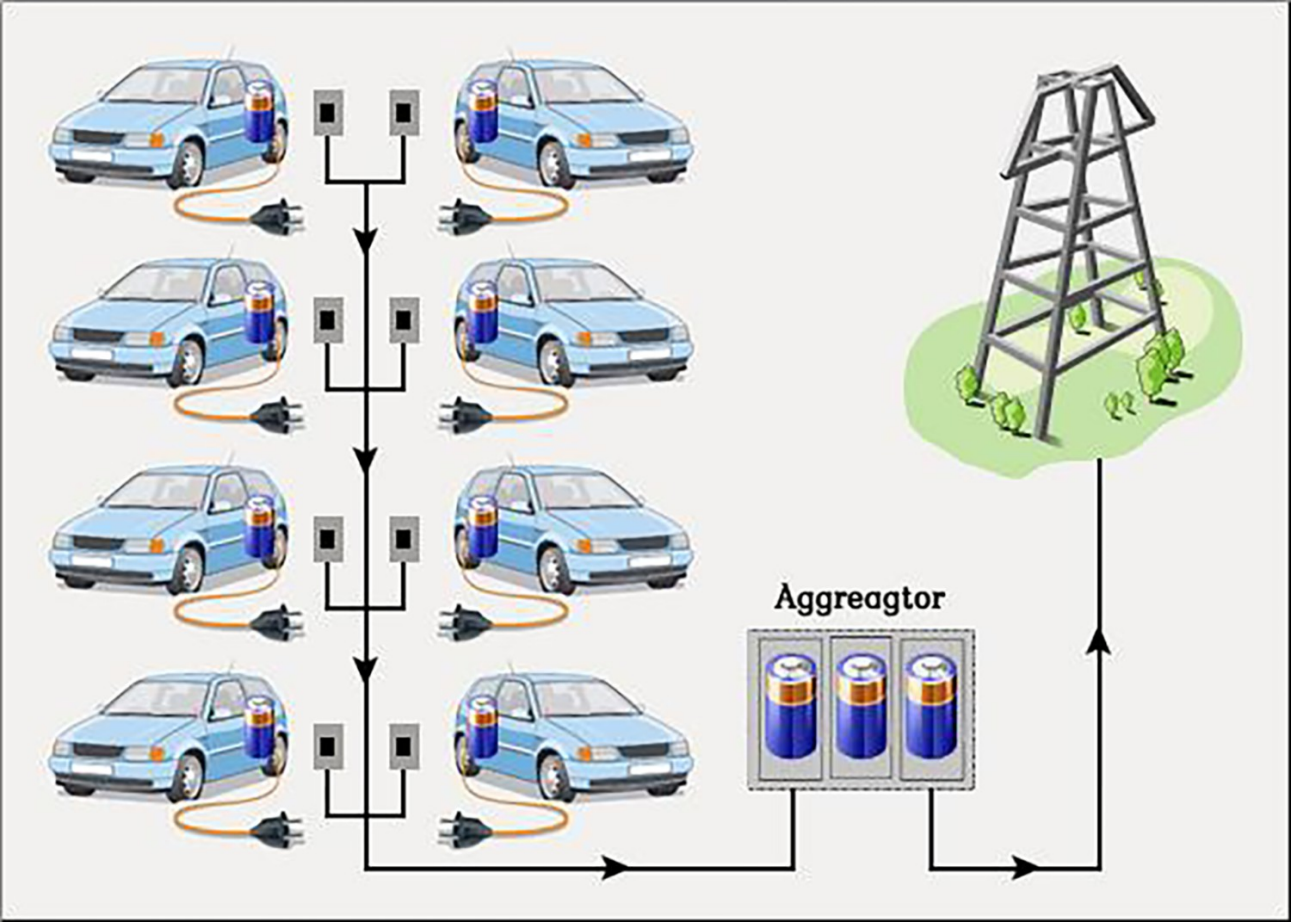
V2G smart scheme.

**Fig 3:**
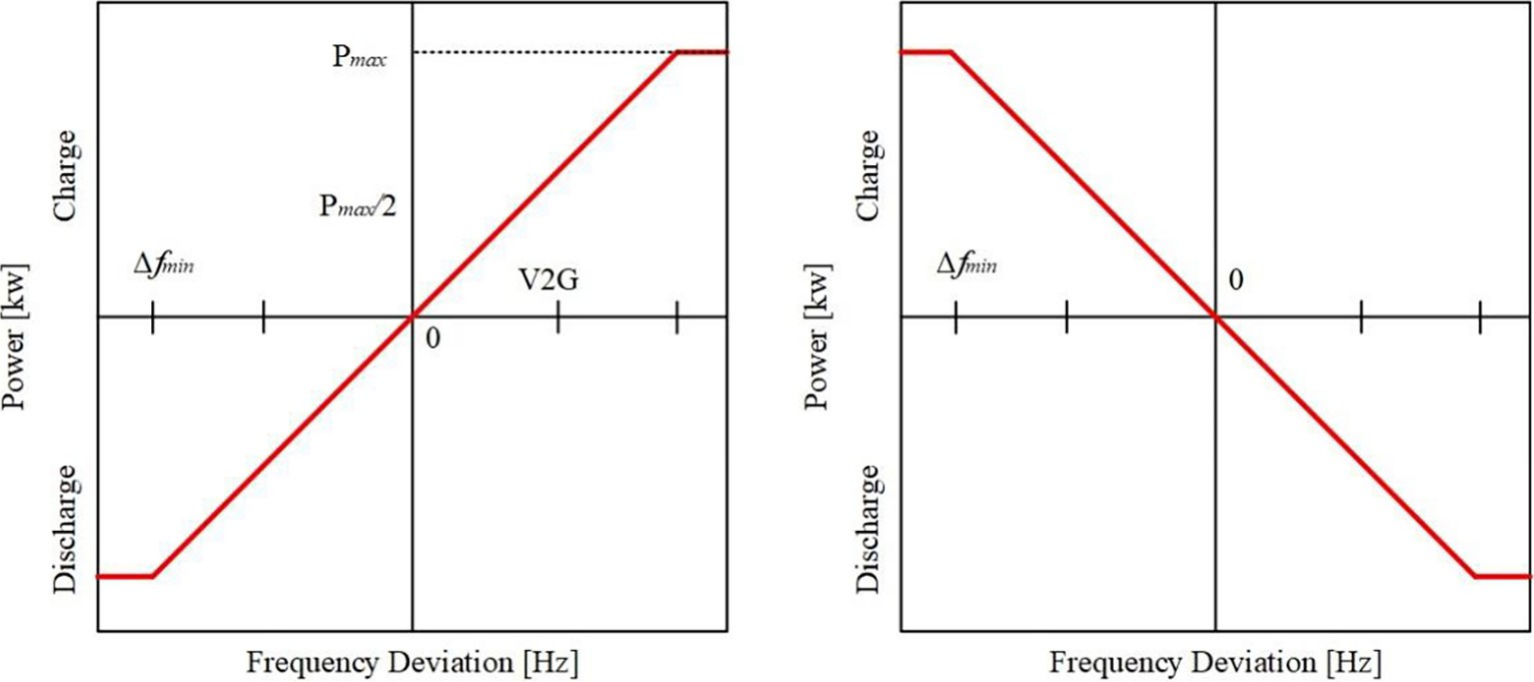
Power/frequency deviation curves of the V2G.

**Fig 4:**
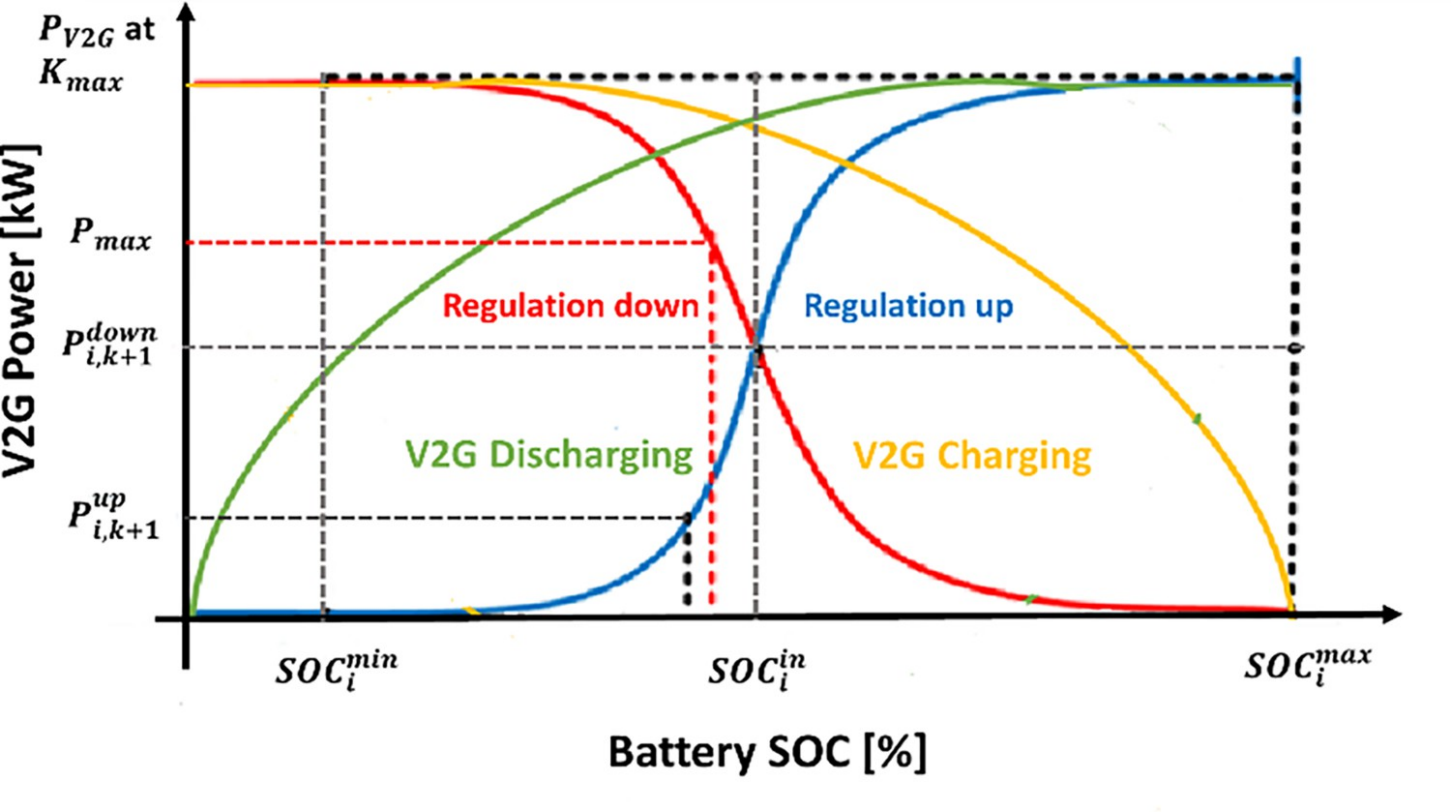
EVs battery SOC balance control considering V2G power.

The V2G smart grids are built on exploiting the EVs during the periods of its plug-in, as illustrated in Fig 2. Without interfering with the EV users’ ability to charge their vehicles; this can be done following a pre-scheduled agreement. V2G is mainly applied in the PS to treat the problems of F-deviations and load changes that result from the presence of RESs like PV. So, the V2G arrangement will improve the response of the system, especially throughout the fluctuations periods in RESs. Fig 3 illustrates the battery output power of the V2G arrangement. The slope of the battery output power is negative in the charging period and positive in discharging period as a result of the F-violation value and sign (Δ*f* = *f*_*actual*_−*f*_*reference*_). The relation between the V2G power and the F-deviation can be defined as described in the following equation [20]. Fig 4 shows the EVs battery SOC balance control considering V2G power, and Table 1 indicates the values of V2G control parameters.

$$P_{V2G}=\left\{ \begin{array}{l} K_{V2G}.\Delta f\;,K_{V2G}.\Delta f<P_{max}\\ P_{max}\;,P_{max}<|K_{V2G}.\Delta f| \end{array}\right.$$ (3)

where K_V2G_ is the gain of the V2G that can be calculated from the following equation with considering charging/discharging battery balance control:

$$K_{V2G}=K_{max}\left\{1-{\left(\frac{SOC-{SOC}_{low/high}}{{SOC}_{max/min}-{SOC}_{low/high}}\right)}^{n}\right\}$$ (4)

where *P*_*eBase*_ equals 800MVA.

**Table 1:** V2G controls parameters [36].

Parameters	Value
V2G maximum power (*P*_*max*_)	40kw, 5kw/battery, 8 batteries
V2G maximum gain(*K*_*max*_)	200kw/Hz
*n*, *SOC*_*min*_, *SOC*_*low*_, *SOC*_*high*_, *SOC*_*max*_	(2,10, 20, 80, 90) %
∆*f*_*min*_	-0.1Hz

## 4. Ecological technique

Optimal control techniques help the controllable system to be robust and stable in the face of various perturbations. One of these methods is an ecological technique using state space perspectives [38, 39]. The idea of the ECO control system depends on the internal interactions of species [40]. Table 2 indicates the four types of ecosystems, and these interactions are (1) Competition, (2) Mutualism, (3) Amensalism/Commensalism, and (4) Predation (Parasitism) [24, 31, 40].

**Table 2:** Types of interactions between two species in an ecosystem.

Type no	Interaction type	Matrix representation
1	Competition	$\left[\begin{array}{cc} {}* & -\\ - & * \end{array}\right]$
2	Mutulism	$\left[\begin{array}{cc} {}* & +\\ + & * \end{array}\right]$
3	Commensalism	$\left[\begin{array}{cc} {}* & +\\ 0 & * \end{array}\right]$
	Amensalism	$\left[\begin{array}{cc} {}* & -\\ 0 & * \end{array}\right]$
4	Predation (Paraslism)	$\left[\begin{array}{cc} {}* & +\\ - & * \end{array}\right]$

Sign stability (Hurwitz stability) investigation is used to test the stability of this ecological model [31]. For ’sign ’stability’, these conditions are necessary for any matrix *T* with entries *t*_*kl*_:

- *t*_*kk*_ < 0 for all *k* < 0,
- *t*_*kk*_ < 0 for one value of *k* or more,
- *t*_*kl*_*t*_*lk*_ < 0 for all k; l where k ≠ l,
- *t*_*kl*_*t*_*lh*_*t*_*mk*_ = 0, for any cycle of three or higher different indices *k*,*l*,…, *m*.
- Det(*T*) ≠.

### 4.1 Mathematical perspective of sign stability

#### 4.1.1 Eigenvalue distribution

The stability and eigenvalue distribution of ecological models must be considered. At sign stable matrices, the eigenvalue distribution along both the imaginary and the real axes can be calculated in a relatively easy method. In time invariant matrix theory, matrices stability is controlled by the nature of the negative real part of its eigenvalues. It is always useful to get bounds on the Eigen value distribution of a matrix with as little computation as possible [41, 42].

A few new results are listed in this aspect.

Nature of Ecological perspective interaction and its role in stability.

Quantitative engineering perspective: sign stable ’matrices’ Eigen value distribution.

There are some theorems for eigenvalue distribution along the real axis which designed according to some observations, such:

Theorem 1

(situation of all diagonal elements are negative):

For all n×n sign stable matrices, with all negative diagonal elements, there are bounds on the real parts of the eigenvalues such as:

""""The minimum magnitude diagonal element can represent the lower bound on the magnitude of the real part while the maximum magnitude diagonal element in the matrix can represent the upper bound" " "".

That is, for an n×n ecological sign stable matrix A = [aij],

$${\left|a_{ii}\right|}_{min}\leq {\left|Re(\lambda )\right|}_{min}\leq {\left|Re(\lambda )\right|}_{max}\leq {\left|a_{ii}\right|}_{max}$$ (5)

Theorem 2

(Case of some diagonal elements being zero):

In the case of zeros values of the ecologically sign stable diagonal matrix, the bounds can be given by:

$${\left|a_{ii}\right|}_{min(=0)}<{\left|Re(\lambda )\right|}_{min}\leq {\left|Re(\lambda )\right|}_{max}\leq {\left|a_{ii}\right|}_{max}$$ (6)

Also, that is for an n×n ecological sign stable matrix *A* = [*a*_*ij*_].

In fact, theorems 1 and 2 minimize the computation for the distribution of eigenvalues. Actually, the distribution can be determined by more observation.

Similarly, the following theorem can be stated for n× n matrix for the imaginary part of the eigenvalues.

Theorem 3

For all n× n ecologically sign stable matrices, bound on the imaginary part of the eigenvalues can be given by:

$${\left|Imag(\lambda_{i})\right|}_{max}=\sqrt{\sum_{i,j=1}^{n}\left(-a_{ij}a_{ji}\right)}\;\forall i\neq j$$ (7)

### 4.2 Design of robust control technique

Considering the following system equation:

x˙=Ax+Bu (8)

*x* refers to n_1_ state vector and the control *u* refers to m_1_. (A, B) should be totally controllable.

### 4.3 Closed loop controller

The following eqution presents the system of linear time invariant (LTI)

x˙(t)=Ax(t)+Bu(t) (9)

And *u* = *G x*, (*G* refers to the controller gain).

**Fig 5:**
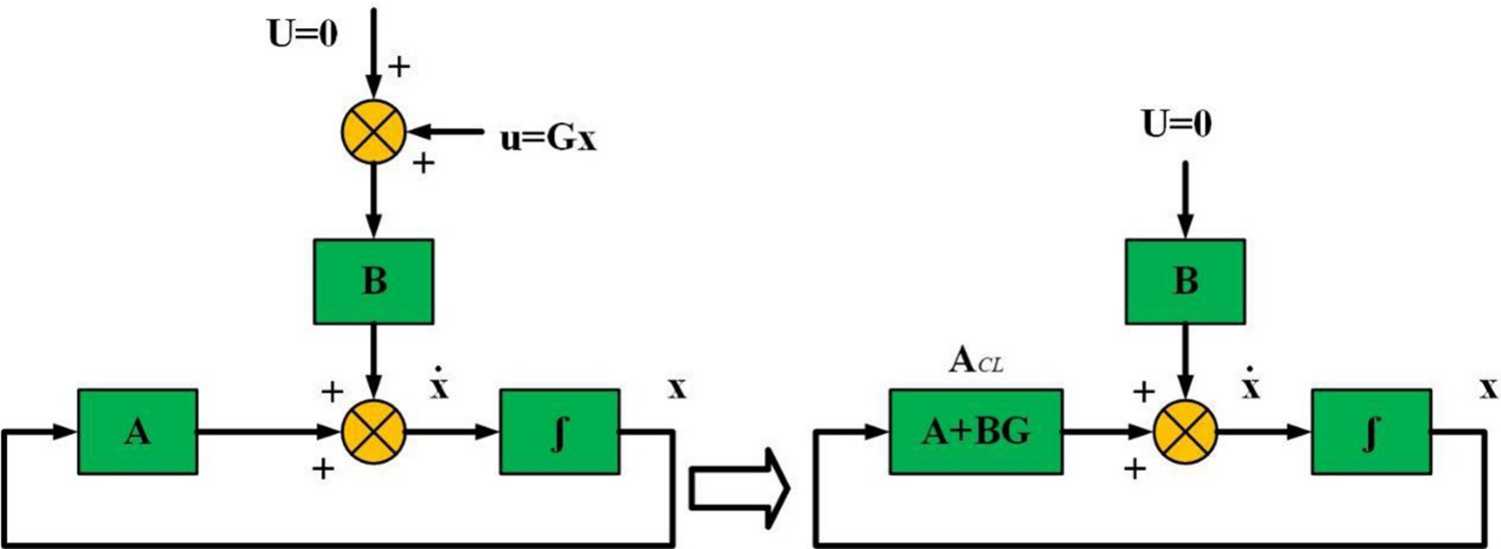
Ecological system.

As indicated in Fig 5, the nominal feedback target of the system matrix could be given by the next equation:

$$A_{cl}=A+B\;G$$ (10)

Firstly, the existence of the control gain G should be tested as in [32], then the closed loop matrix *A*_*cl*_ should be determined as in [37], and finally, G should be determined as in [34, 43]. *G* is positively related by the controllability of (A, B). Also, in choosing *A*_*cl*_, many pure predator–prey links beside several negative diagonal elements should be considered.

For the asymptotically stable case of *A*_*cl*_, the following equation can describe this case:.

$$G=B^{-1}(A_{cl}-A)$$ (11)

where,

$$G=\frac{{-R}_{o}^{-1}B^{T}K}{\rho_{c}}$$ (12)

And using the following Riccati equation

$$KA+A^{T}\;K-KB\frac{R_{o}^{-1}}{\rho_{c}}B^{T}\;K+Q=0$$ (13)

Then *G* in Eq (9) represents the Riccati-based control gain, *K* is the positive definite solution, *R*_*o*_ and *Q* have been calculated as positive symmetric definite weighting matrices, *ρ*_*c*_ acts as the design variable. The ecological model’s eigenvalues distribution and stability should be in consideration. So, the negative real part nature of the matrix eigenvalues should control its stability. Bound of the eigenvalue distribution of a matrix is needed.

Kalman filter observer has been utilized for the estimation of the total states of the system, these states are needed for the optimal state feedback *u* = *G x*. Only *∆f*_*i*_, *∆P*_*tie*_, *i*, and *∆P*_*ci*_, are applied as inputs of the filter.

The following equation is used to drive the ’ ’system’s states

$$(\hat{\dot{x}})=(A-BK-LC){\hat{x}}_{i}+LY$$ (14)

where *L* refers to the gain of the Kalman filter. This gain could be chosen by calculating the system noise and measurement covariance *Q*_*n*_ and *R*_*n*_. Also, the Kalman filter is suitable for linear systems [32].

## 5. Ecological controller for three area LFC power system

**Fig 6:**
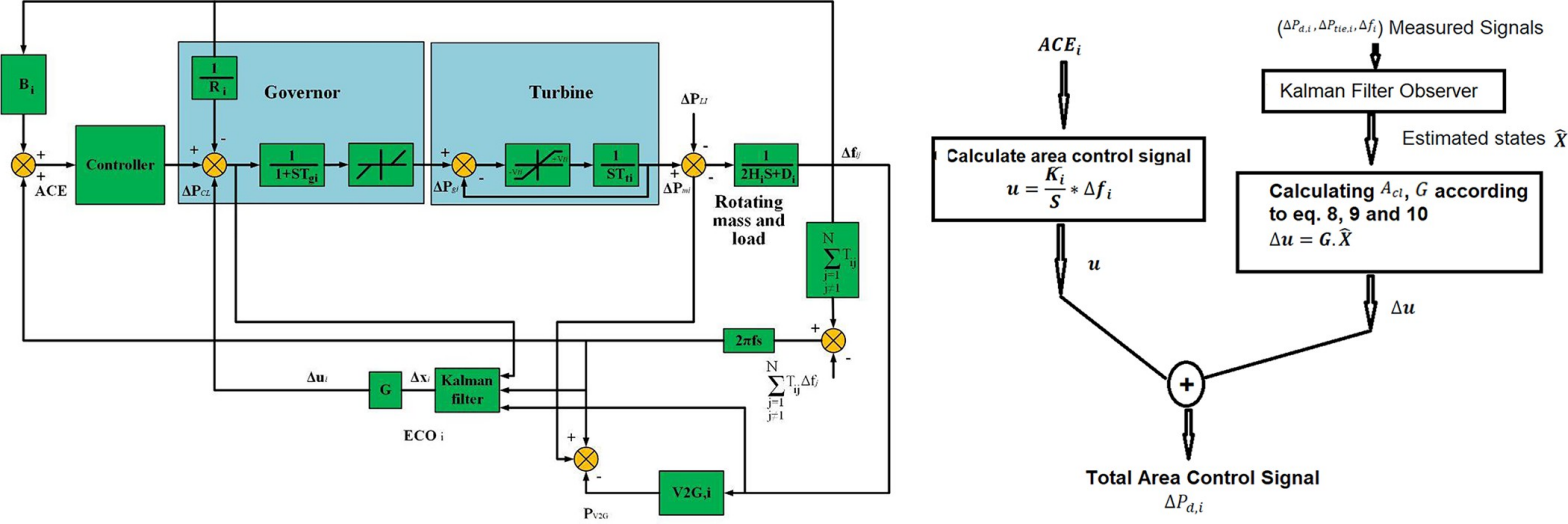
**(a)** Interconnected power system involving the suggested (I + ECO +V2G) scheme. **(b)** the flow chart of the proposed control method.

**Fig 7:**
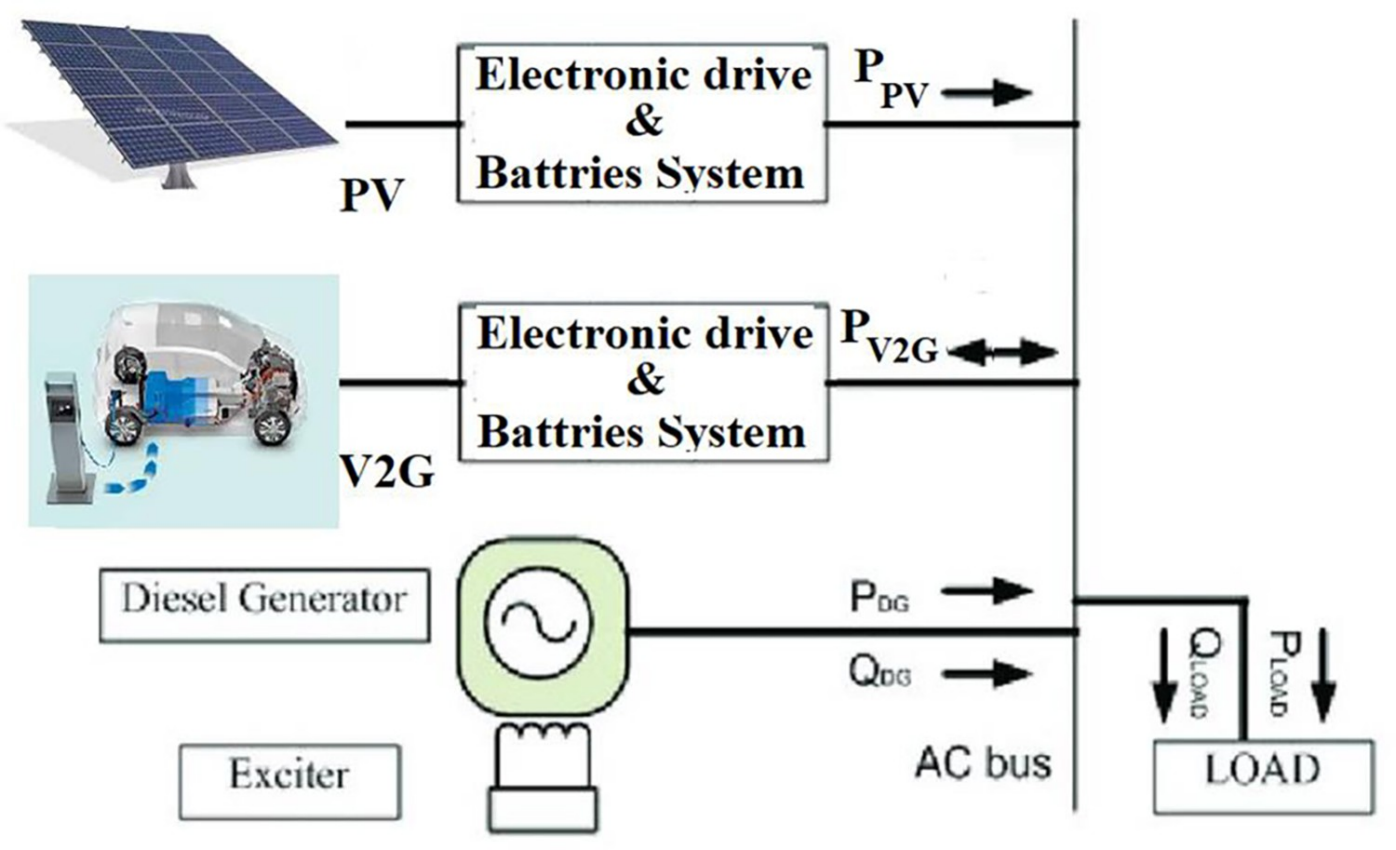
A schematic of the proposed model with power flow.

The multi-area power system will be adjusted, as illustrated in Fig 6, by taking (*i* = 1, 2, 3) to be a three-area power system, and it is used as an examination system. As shown in Fig 6A, GRC and speed governor with dead-band constraint are deemed [37, 44]. In addition, Fig 6B illustrates the flow chart that describes the procedures sequences of the proposed control technique The system controller has been designed in a decentralized manner; every local area controller has been designed individually. Furthermore, the Kalman filter is applied in every area to assess the system ${\hat{x}}_{i}=[\Delta {\hat{f}}_{i}\;\Delta {\hat{P}}_{mi}\;\Delta {\hat{P}}_{gi}\;\Delta {\hat{P}}_{tie,i}]$ which have been multiplied by *ECO* feedback gain to provide the additional optimal control signal. In addition, *∆f*_*i*_ is applied to the V2G system to give the V2G power which is added to *∆P*_*mi*_. A schematic of the proposed model with power flow is depicted in Fig 7.

## 6. Results and discussions

**Fig 8:**
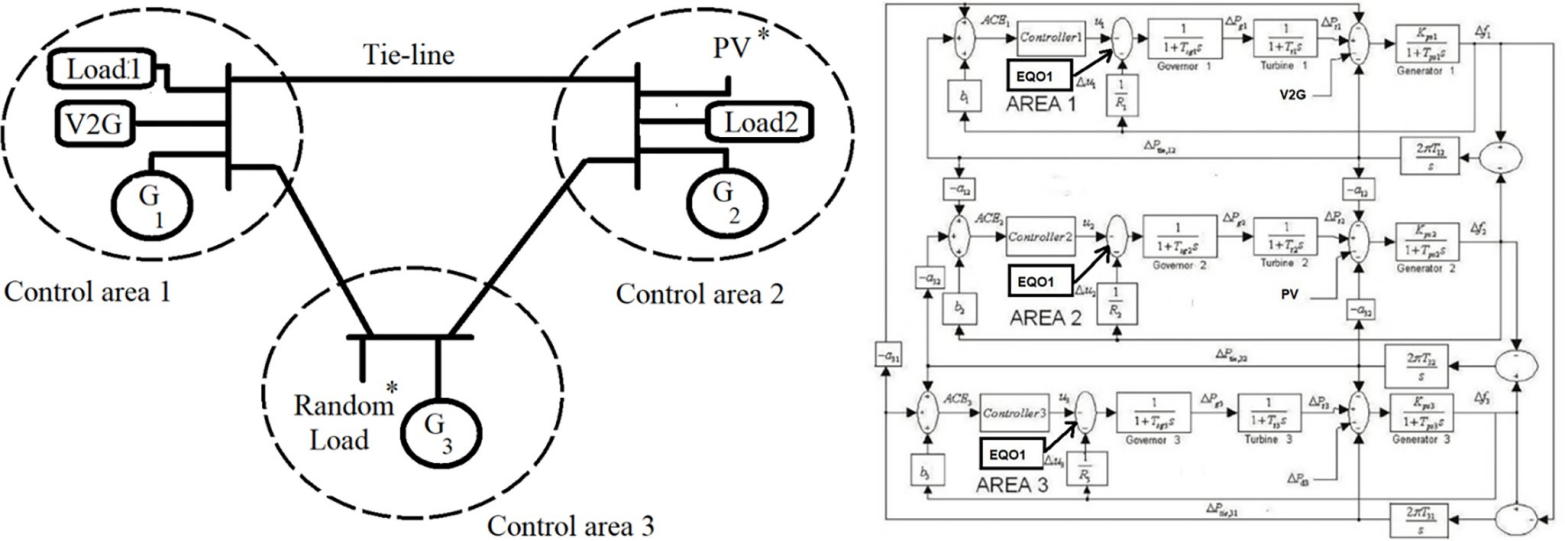
Studied interconnected systems.

The ecological controller is executed on a three-area interconnected power system by finding its state space model. MATLAB/Simulink as a simulation tool has been exploited to confirm the accomplishment of the suggested control scheme. Fig 8A shows the three-are controlled power system interconnected together for this purpose. Table 3 demonstrates the simulation parameters for each area used in the studied system [37]. In addition, Fig 8B illustrates the block diagram of the proposed three-area interconnected power system. In addition, only in Area-1, a 12 MW V2G system (300 cars with detailed parameters listed in Tables 1 and 4 is considered in the studied cases. As a prescheduled agreement, all vehicles should be plugged in from 11 pm until 7 am by their owners.

**Table 3:** Practical data and parameters of a three-controlled area of power system [37].

Area	*K*(*s*)	*D*(*p*.*u*/*Hz*)	2*H*(*pu*.*sec*)	*R*(*Hz*,*pu*)	*T*_*g*_(*sec*)	*T*_*t*_(*sec*)	*Tij*
Area-1	-0.3/s	0.015	0.1667	3.00	0.08	0.40	*T*_12_ = 0.20, *T*_13_ = 0.25
Area-2	-0.2/s	0.016	0.2017	2.73	0.06	0.44	*T*_21_ = 0.20, *T*_23_ = 0.15
Area-3	-0.4/s	0.015	0.1247	2.82	0.07	0.30	*T*_31_ = 0.25, *T*_32_ = 0.15

**Table 4:** Battery model specifications.

Parameter	Value
Rating voltage (*V*_*nom*_)	345.6V
Energy Capacity (*C*_*nom*_)	5.2kwh
Internal resistance (*R*_*int*_)	0.4Ω
Current efficiency (*η*)	1.0

In the simulation studied cases, the GRC and the maximum dead band value of the governor are set as 10% per minute and 0.05 p.u. for every area, respectively [45, 46]. These values are used with the proposed control method.

**Fig 9:**
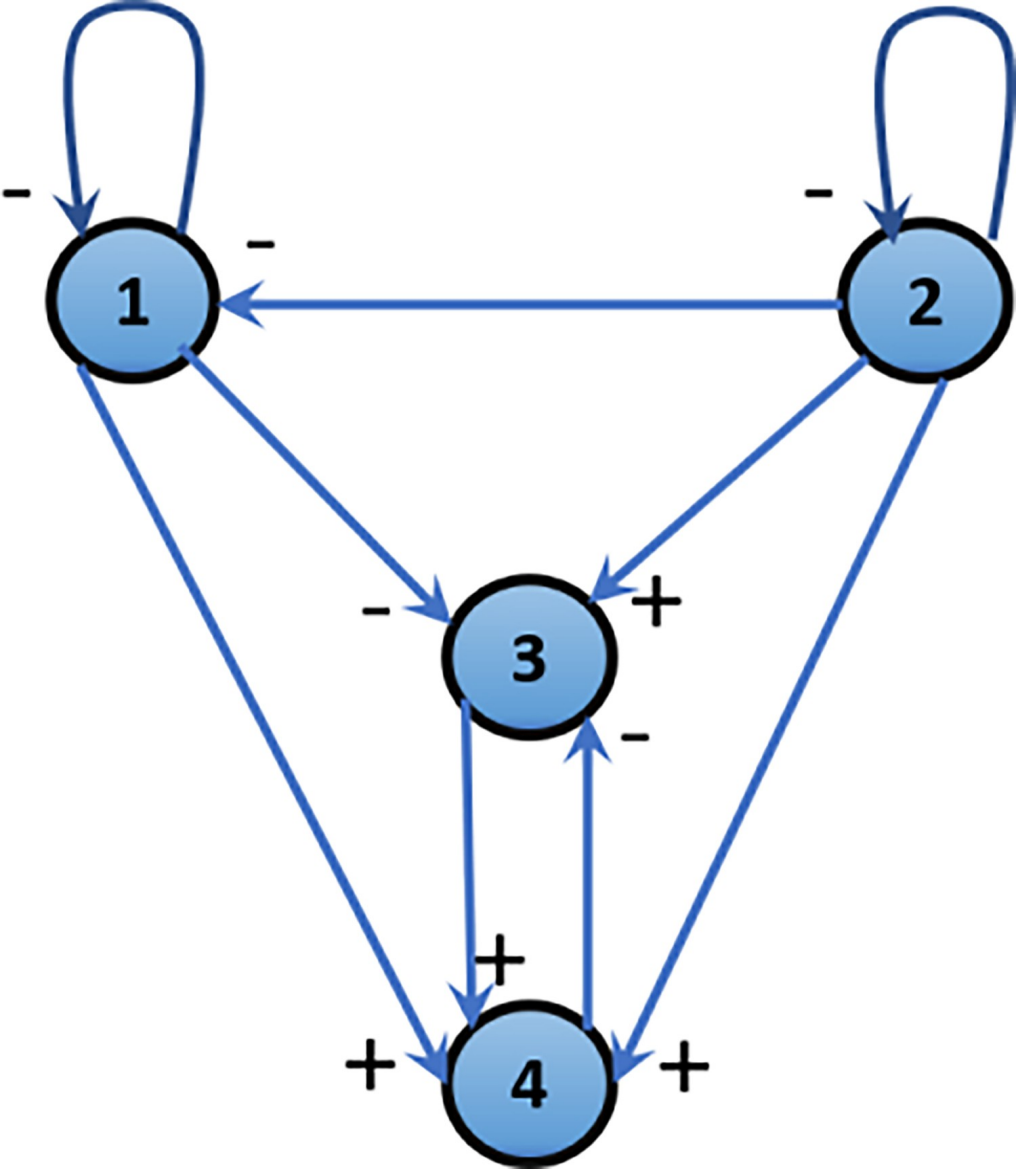
Three area sign pattern matrix corresponding digraph.

The ecological control parameters are given as the following: The pattern matrix of the ecological sign has been chosen as the following:

$$A_{cl,i}=\left[\begin{array}{c} -\;-\;0\;0\\ 0\;-\;0\;0\\ -\;+\;0\;-\\ +\;+\;+\;0 \end{array}\right]$$

where *©* = 1, 2, and 3, and its corresponding digraph is shown in Fig 9.

So, according to the pattern matrices of the ecological sign, the target (nominal closed-loop matrices) can be formed as follows:

$$A_{cl,1}=[\begin{array}{cccc} -5.0 & -6.75 & 0.0 & 0.0\\ 0.0 & -5.0 & 0.0 & 0.0\\ -1.5 & 2.0 & 0.0 & 0.0\\ 5.0 & 1.0 & 8.0 & 0.0 \end{array}]$$

$$A_{cl,2}=[\begin{array}{cccc} -3.0 & -6.75 & 0.0 & 0.0\\ 0.0 & -3.0 & 0.0 & 0.0\\ -1.5 & 2.0 & 0.0 & 0.0\\ 5.0 & 1.0 & 8.0 & 0.0 \end{array}]$$

$$A_{cl,1}=[\begin{array}{cccc} -4.0 & -6.75 & 0.0 & 0.0\\ 0.0 & -4.0 & 0.0 & 0.0\\ -1.5 & 2.0 & 0.0 & 0.0\\ 5.0 & 1.0 & 8.0 & 0.0 \end{array}]$$

$$G_{1}=\left[\begin{array}{c} 0.6391\;-0.4642\;0.3333\;0.0000\\ 0.1980\;0.5890\;-0.0150\;0.3336\\ -0.8952\;-0.3074\;-0.8232\;0.0000\\ 0.31250.46580.0000\;0.0000 \end{array}\right]$$

$$G_{2}=\left[\begin{array}{c} 0.7944\;-0.6212\;0.3663\;0.0000\\ 0.2605\;0.5530\;-0.0160\;0.6136\\ -0.8621\;-0.2122\;-0.9532\;0.0000\\ 0.2083\;0.1667\;0.0000\;0.0000 \end{array}\right]$$

$$G_{3}=\left[\begin{array}{c} 0.6900\;-0.5575\;0.3546\;0.0000\\ 0.1732\;0.7367\;-0.0150\;-0.0240\\ -0.8311\;-0.1945\;-0.9032\;0.0000\\ 0.1111\;0.1111\;0.1111\;0.0000 \end{array}\right]$$

This design can be applied to large-scale systems with multiple RES using the same past steps by determining the dimensions of the system matrix, the value of *A*_*cl*,*i*_ and *G*_*i*_ can be determined.

First, this research will examine the effects of plugging in V2G on the load frequency without any control and to charge only. Then the proposed controller will be used to regulate the frequency using the E2V technique. The ability of the (I+ECO+V2G) control scheme to support the load frequency during these disturbances will be evaluated using three test cases. Those are step changes in the load, variations in the system parameters, and random changes in the load with variations in the amount of PV power generated.

### (a) Impact of plugging V2G on the frequency

**Fig 10:**
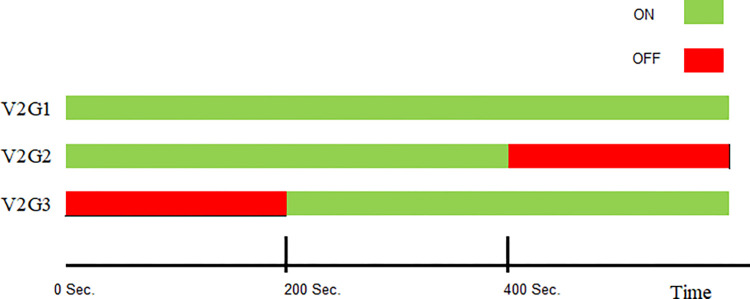
Time plan of plug-in/plug-out of the V2Gs units.

**Fig 11:**
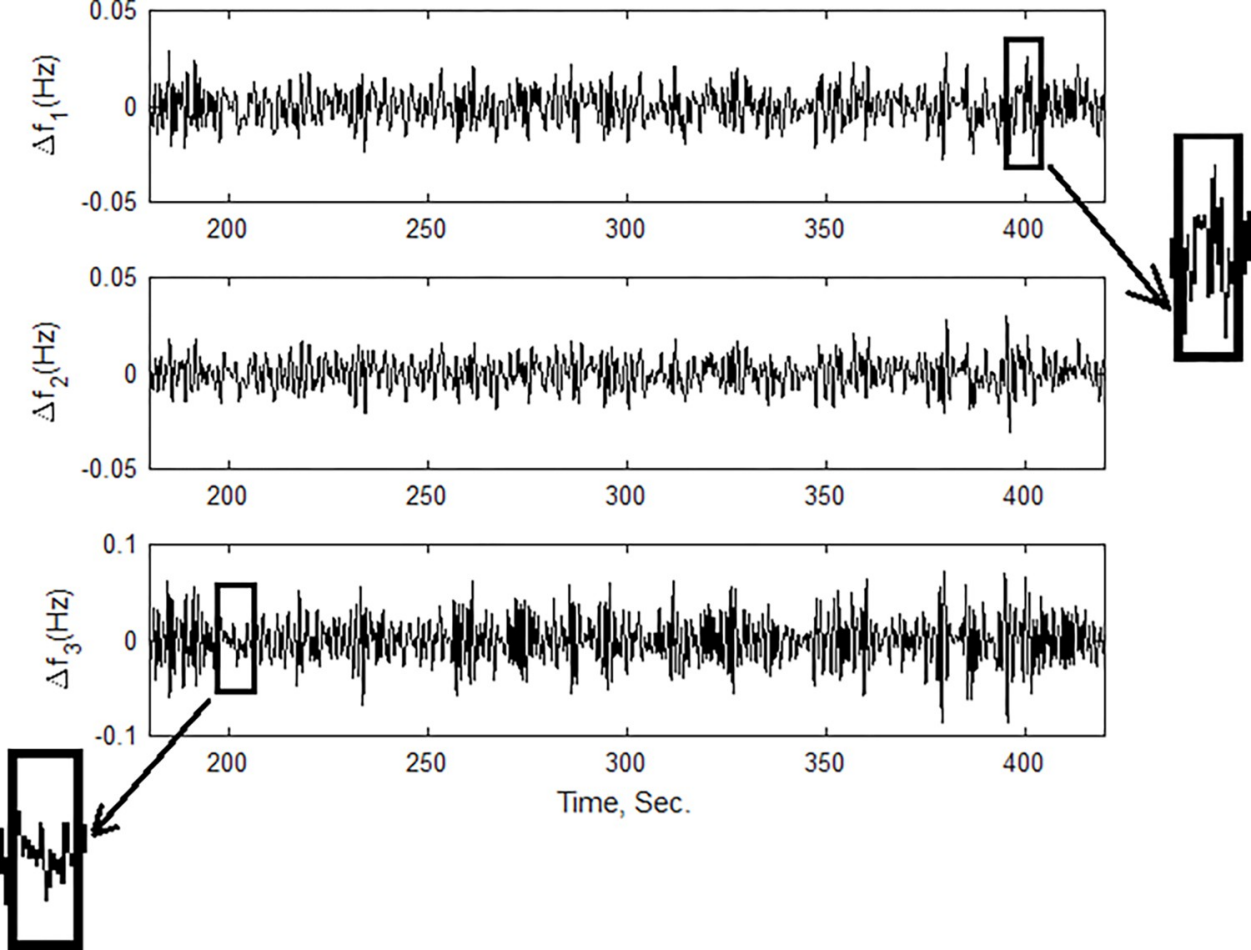
Frequency responses when plugging V2G.

The impact of plugging an EV into the power system on the load frequency profile will be explored in this case. The total V2G unit used in past cases has been divided into three small units; each unit has 4 MW (100 cars), the time plane of plug-in/plug-out of each unit is shown in Fig 10. The load frequency areas 1 and 2 show ripples of 5%. In contrast, the third area shows ripples of 10%, as depicted in Fig 11. This discussion showed that the V2G will negatively affect the system load frequency if it is not controlled.

This study aims to provide an I + ECO + V2G controller scheme for load frequency control a three-area power system with a PV system in one area.

### (b) Case 1: A step change in the load

**Fig 12:**
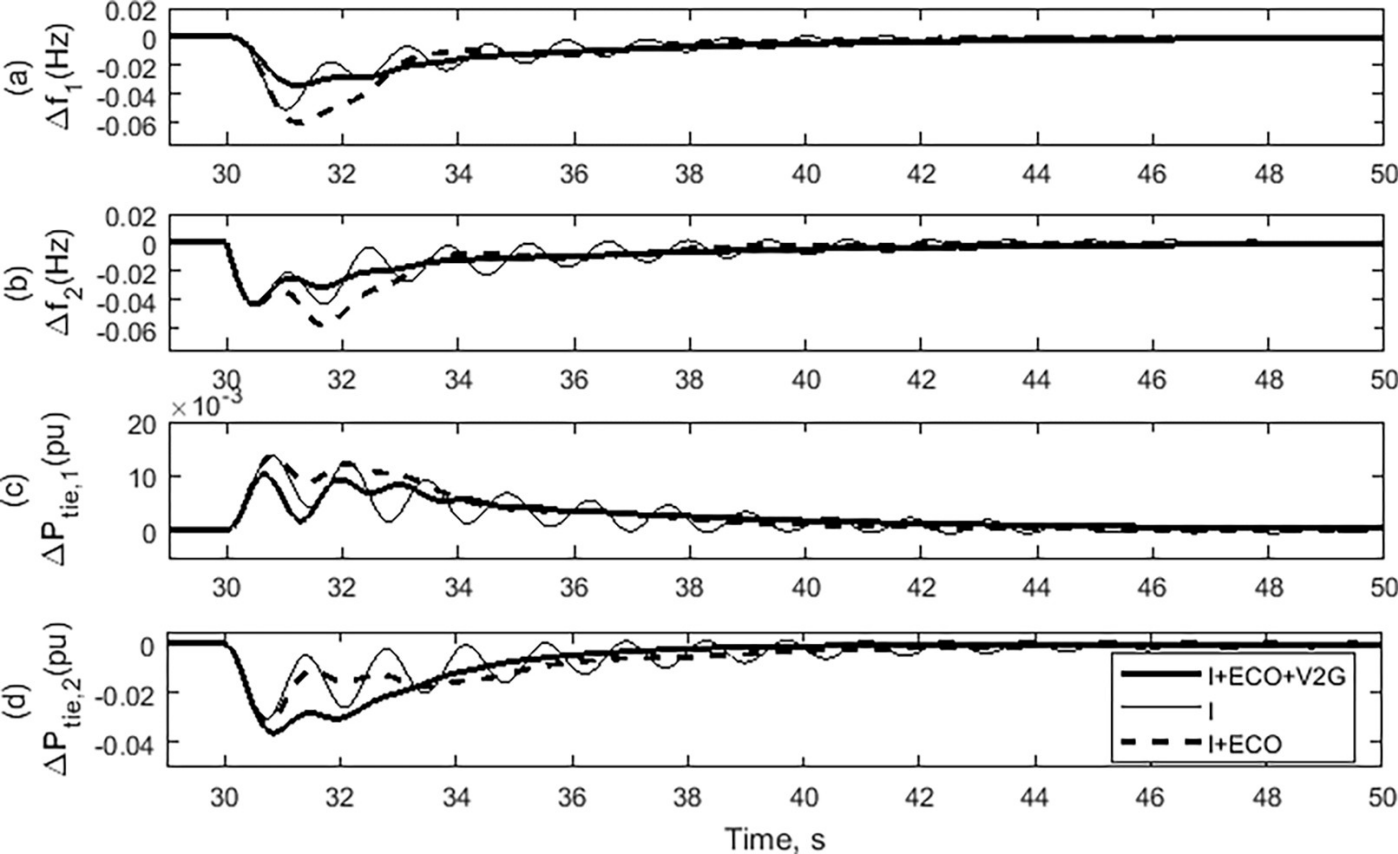
System response of the examined power system in case 1.

In this case study, the effectiveness of the proposed load frequency controller will be assessed by considering the impact of a step change in the load while keeping the system parameters constant. The performance of the suggested control strategy (I + ECO + V2G) is evaluated on the assessed power system by adjusting the load power in Area-2 alone. It will be compared to the performance of the traditional integrator (I) control technique as well as the performance of the (I + ECO) control approach. Fig 12 compares the suggested controller (I + ECO + V2G) and both the I + ECO and the conventional I during a step changing of load power in Area-2 (*∆P*_*L2*_) of 0.02 p.u. at 30.0 sec. The frequency deviation in Area-1 and Area-2 with all control techniques are demonstrated in Fig 12(A) and 12(B), respectively. The change in the tie-line power of Area-1 and tie-line power of Area-2 are illustrated in Fig 12(C) and 12(D), respectively. It can be noticed that the studied power system is more stable and has fewer oscillations by implementing both the proposed (I + ECO + V2G) and (I + ECO) control techniques compared with the conventional integrator (I) control technique. Also noteworthy from Fig 11, the proposed (I + ECO+ V2G) control method is faster than the (I + ECO) control method to reach the steady state.

### (c) Case 2: Changing the system parameters

**Fig 13:**
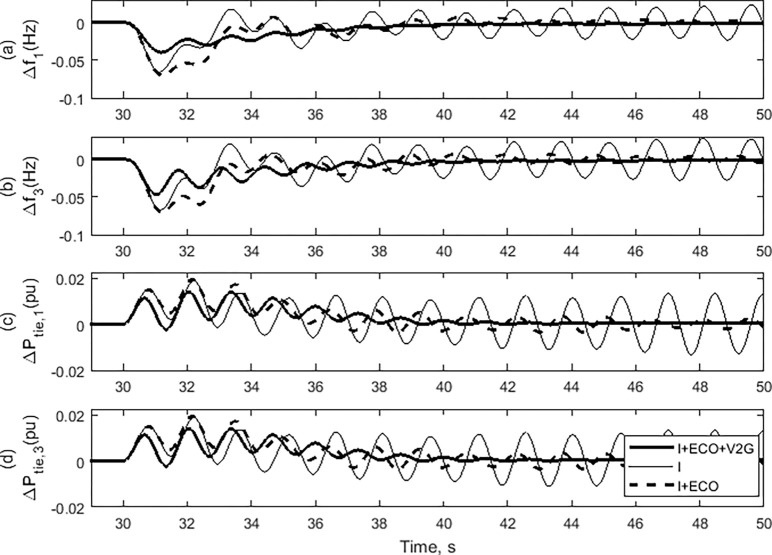
System response of the examined power system in case 2.

The present research aims to assess the influence of several system characteristics on the efficacy of the proposed I + ECO + V2G controller in regulating frequency. The time constants of the governor and the turbine have been increased, according to Table 5. In this case, Fig 13 compares the outcomes of the three control techniques utilized in this study with the suggested changes in the governor and turbine time constants.

**Table 5:** Increasing the time constants parameters of the turbine and governor.

Areas	*T*_*g*_ (*sec*) old	*T*_*g*_ (*sec*) new	Percentage of increasing (%)	*T*_*t*_ (*sec*) old	*T*_*t*_ (*sec*) new	Percentage of increasing (%)
Area-1	0.08	0.105	31	0.40	0.785	96
Area-3	0.07	0.150	114	0.3	0.7	133
Area-3	0.07	0.150	114	0.3	0.7	133

The performance of the examined power system when employing the traditional integrator (I) controller is unstable. Moreover, it has substantially worsened as a result of the changes in the turbine and ’ ’governor’s time constant parameters for the frequency deviations of Area 1 and 2, respectively as depicted in Fig 13(A) and 13(B). The frequency fluctuates reaches a value greater than 5%, which was outside of acceptable limits [47]. Compared to I + ECO, the proposed I + ECO + V2G controller stabilized the investigated power system more quickly and with less vibration. The tile line power of Areas 1 and 2 are plotted in Fig 13(C) and 13(D), respectively. The proposed controller exhibited less severe oscillations than the other controllers, almost bringing this power to zero.

**Fig 14:**
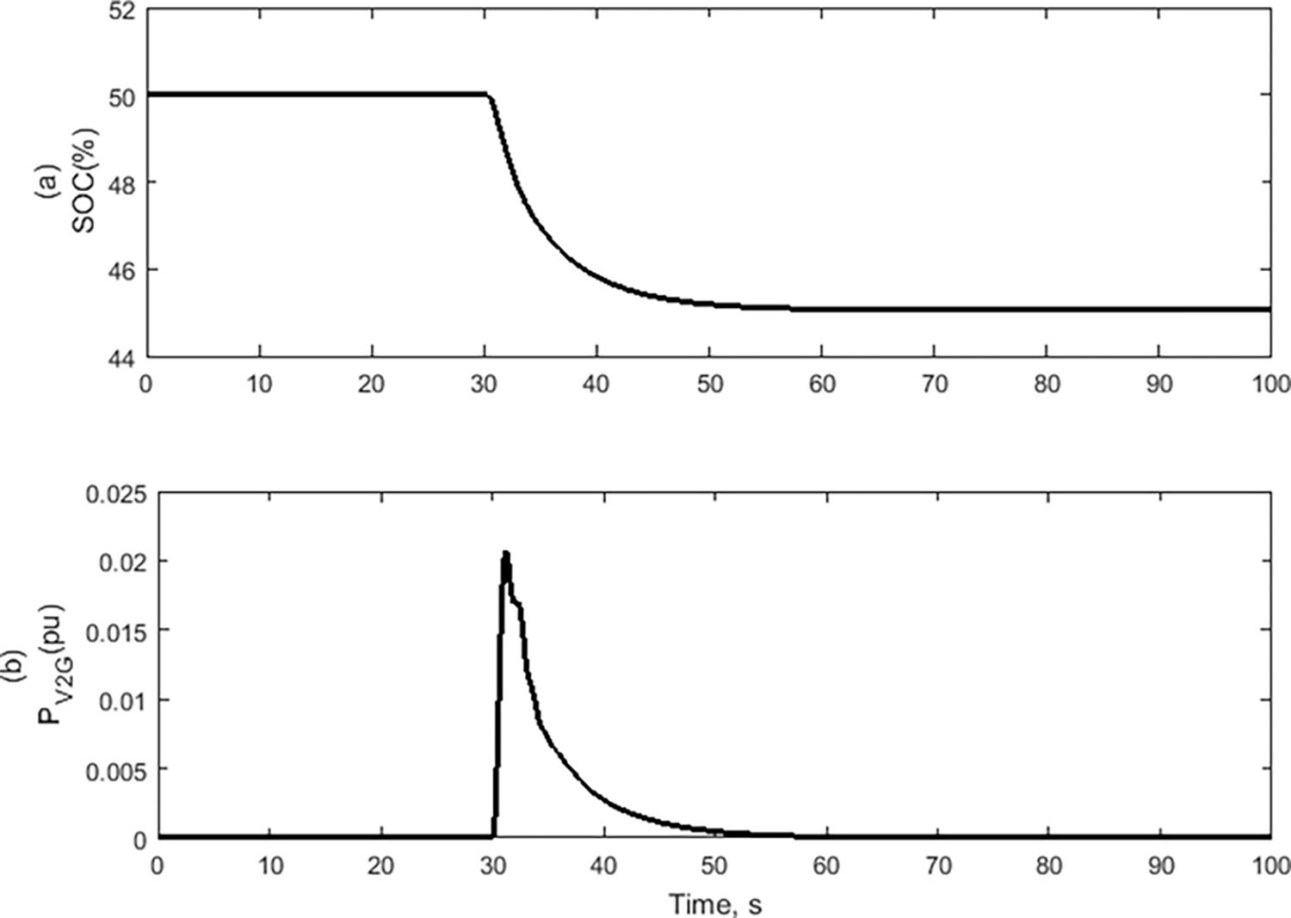
V2G Signals: (a) Percentage of state of charge of one Battery, (b) total output power of V2G system.

The V2G batteries provide power to the system when the generator and governor time constants vary. Consequently, the SOC decreases to regulate the system load frequency as indicated in Fig 14.

### (d) Case 3: Random load changes and PV power variation

In this case, the system has been tested under both random load change in Area-3 and fluctuation of the PV power generated (100 MVA PV system in Area-2). The uncertainties of PV are considered, and the combined uncertainty for the module performance ratio module performance ratio (MPR) [41, 42]:

$$u_{MPR}=\sqrt{\left(u_{fOAI}^{2}+u_{fspectral}^{2}+u_{fG}^{2}+u_{fT}^{2}\right)}$$

where u is the relative standard uncertainty for each influencing factor.

**Fig 15:**
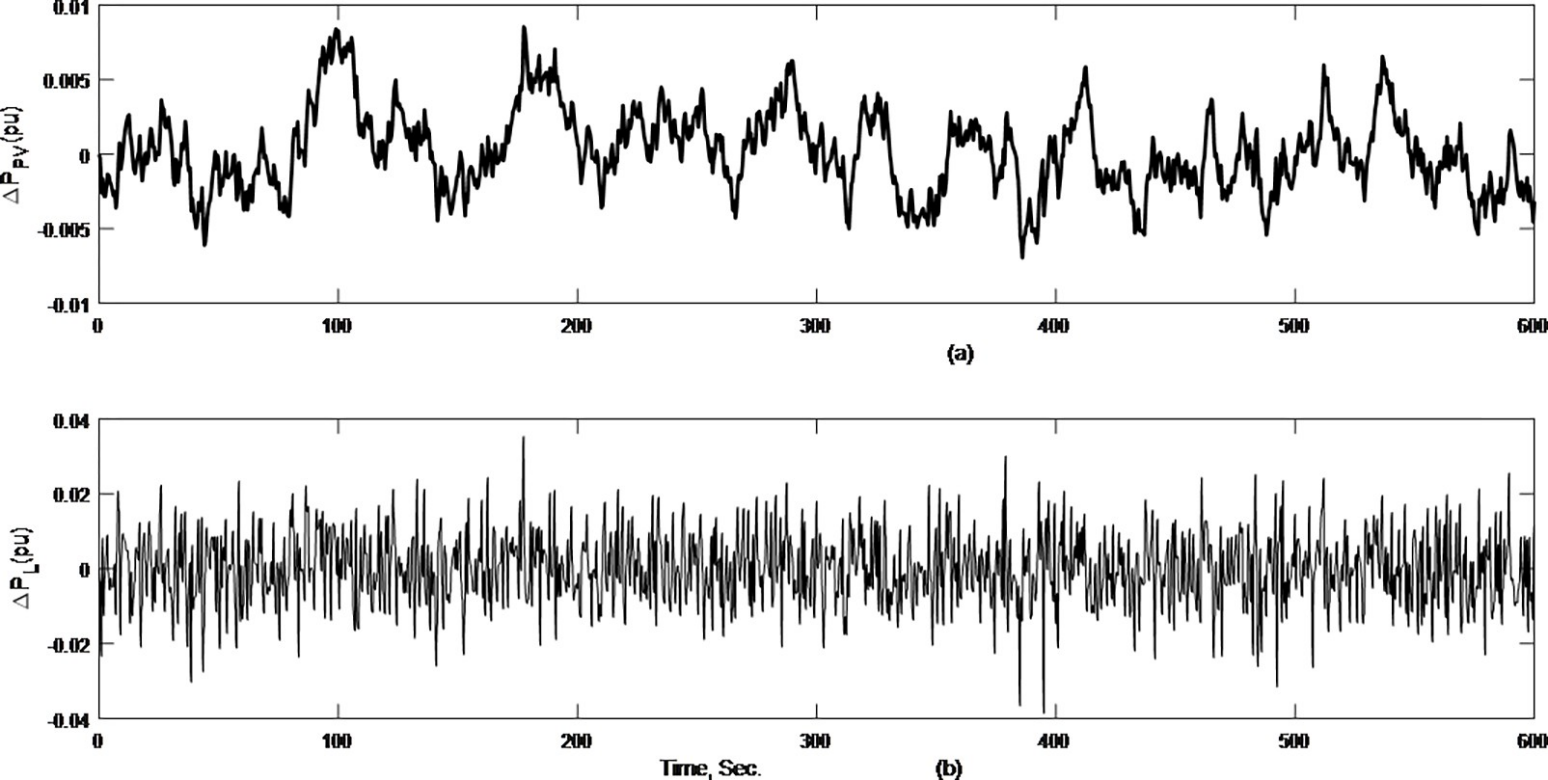
Fluctuation sources for case 3.

**Fig 16:**
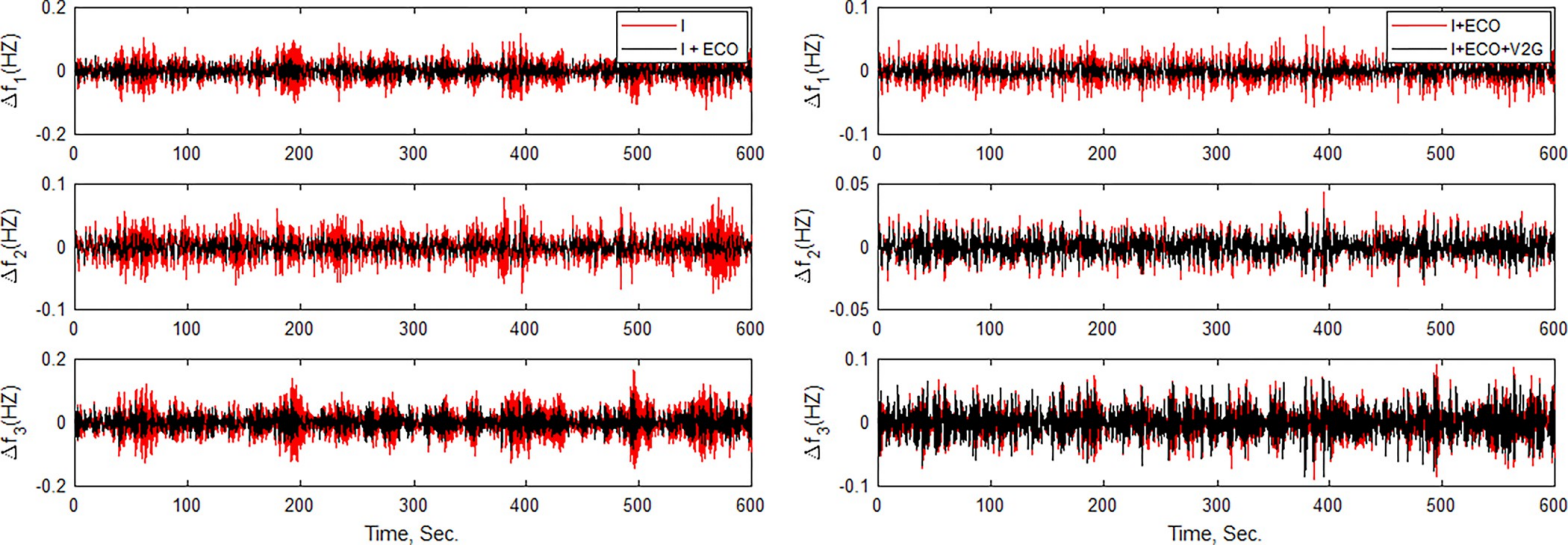
Frequency responses for case 3.

The random load profile and PV power variations are shown in Fig 15. Regarding system load frequency; the recommended I+ECO+ V2G controller scheme performs better than the two alternative techniques (I and I+ECO) as illustrated in Fig 16.

### (e) Real-time implementation

**Fig 17:**
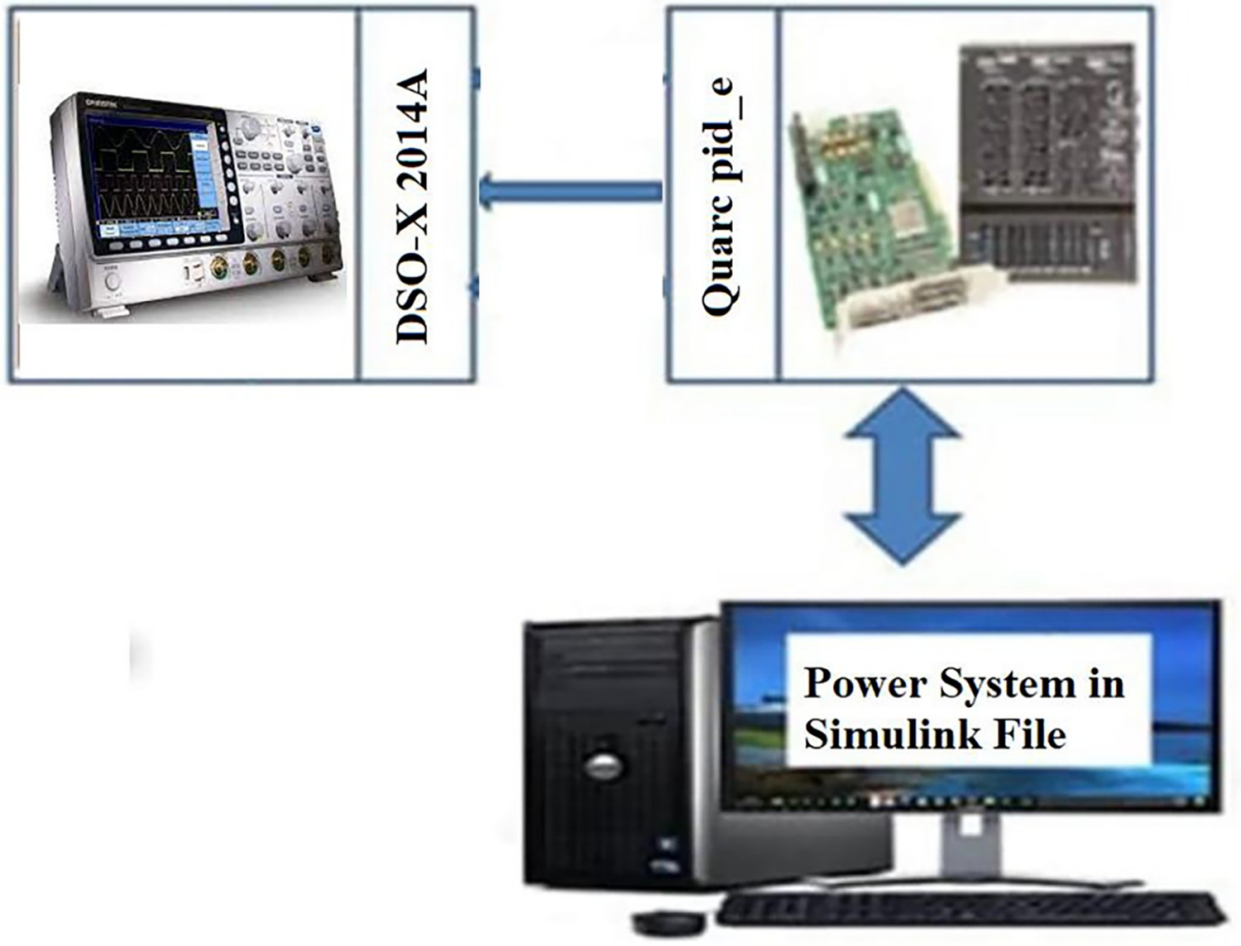
Block diagram of the studied system using RTS.

**Fig 18:**
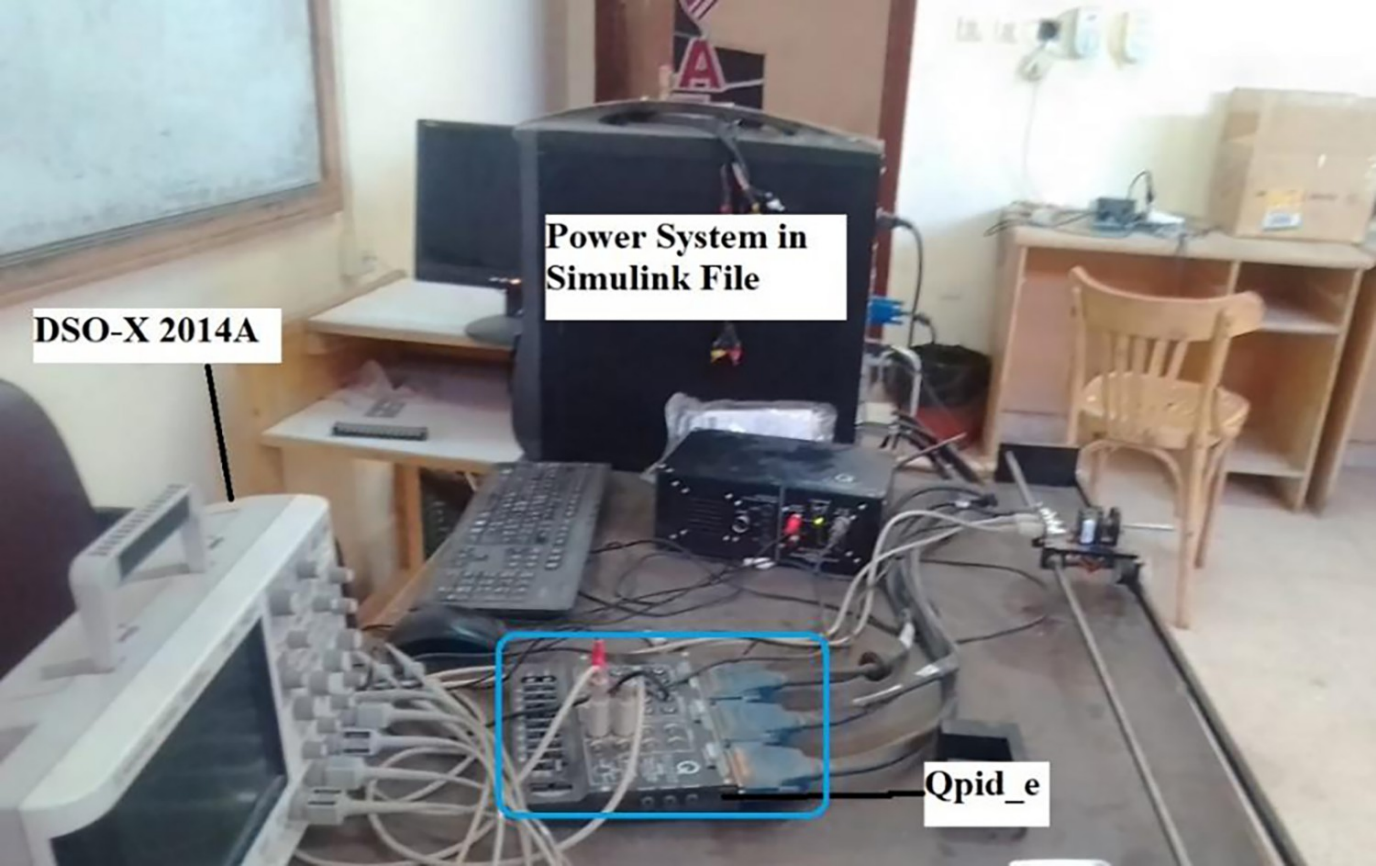
Real-time laboratory setup.

**Fig 19:**
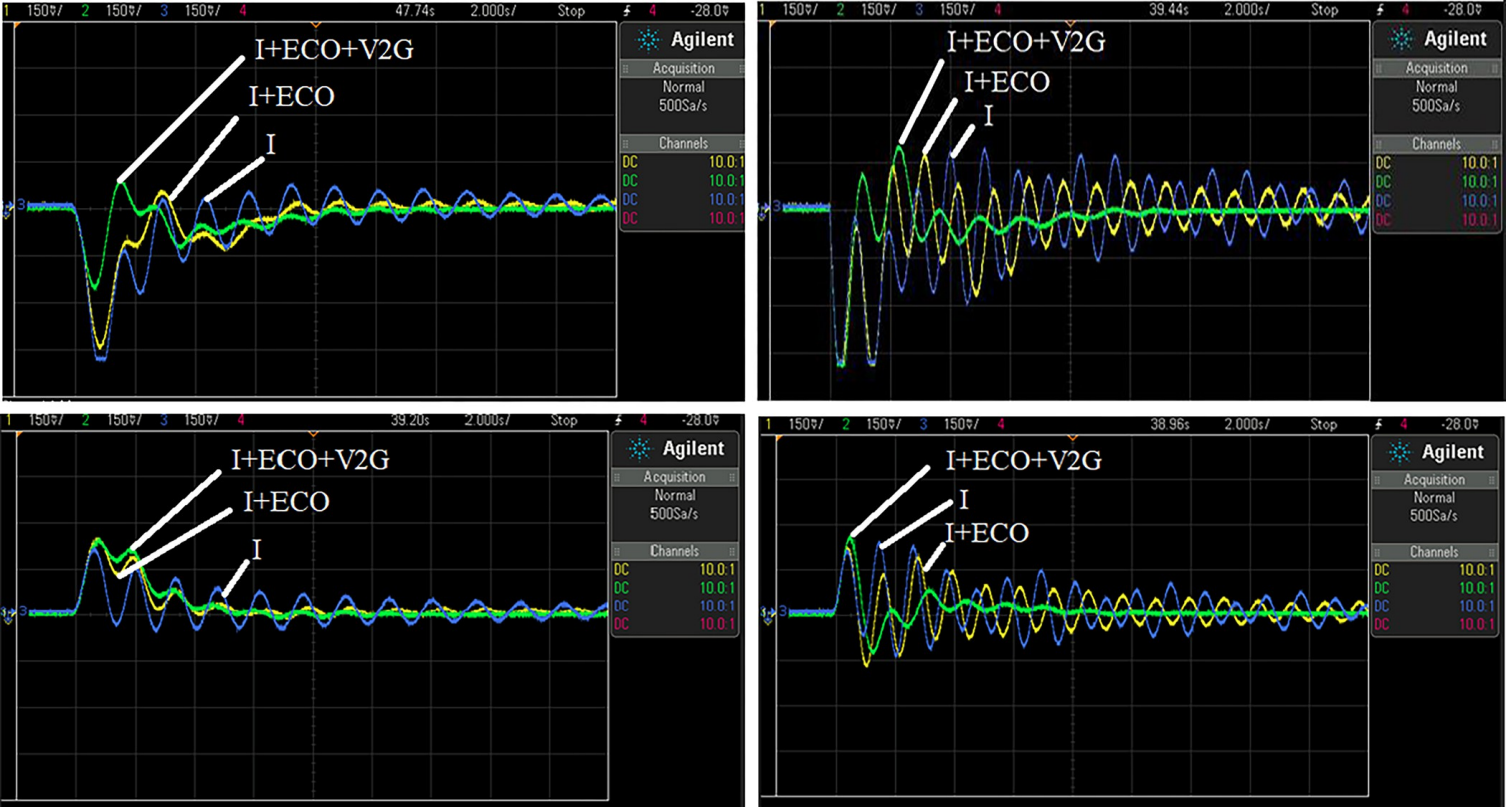
**(a).** The effect of changing in the turbine and ’ ’governor’s time constant parameters for the F-deviations of Area 1 in RTS. **(b).** The effect of changing in the turbine and ’ ’governor’s time constant parameters for the F-deviations of Area 3 in RTS. **(c).** The effect of changing in the turbine and ’ ’governor’s time constant parameters for tile line power of Area 1 in RTS. **(d).** The effect of changing in the turbine and ’ ’governor’s time constant parameters for tile line power of Area 3 in RTS.

**Fig 20:**
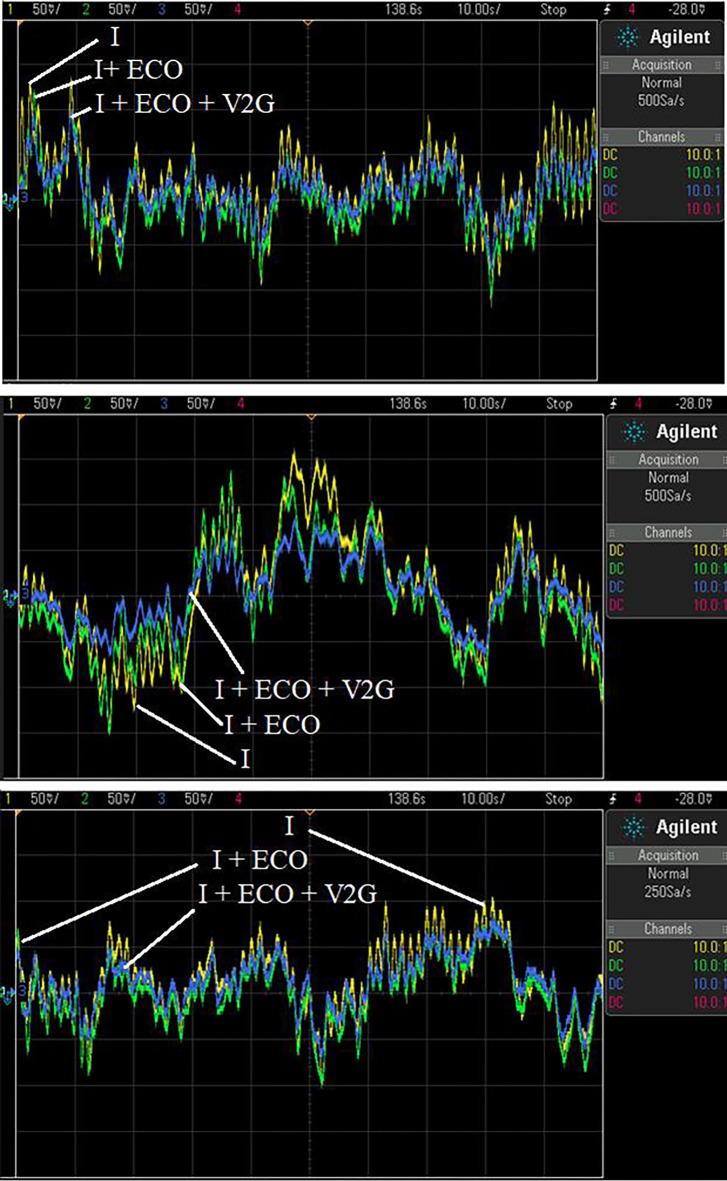
**(a).** The frequency change (*∆f*_*1*_) response with different control techniques of Area 1 in RTS. **(b)**. The frequency change (*∆f*_*2*_) response with different control techniques of Area 2 in RTS. **(c).** The frequency change (*∆f*_*3*_) response with different control techniques of Area 3 in RTS.

To evaluate the MATLAB/SIMULINK simulation outcomes for the proposed LFC, case 2 was recreated using the RTS. The studied three-area power system with the proposed controller is embedded on a PC with QUARC pid_e data acquisition card, as shown in Fig 17. The output frequency signals have been recorded using DSO-X 2014A storage oscilloscope. The physical setup of the studied system in real-time simulation environment is illustrated in Fig 18. Fig 19 shows the system output frequency signals in the same situation of the past case 2. Also, Fig 20 illustrates the system output frequency signals in the same situation as in case 3. All RTS proves the effectiveness of the proposed controller compared to the other studied controllers.

## 7. Conclusions

Initially, an examination was conducted to assess the influence of implementing V2G technology on load frequency. The results indicated that, without proper control mechanisms, the implementation of V2G had an adverse effect. Then an I+ECO+V2G control scheme was proposed and used in this article as a load frequency controller for an integrated three-area power system. The effectiveness of the proposed I+ECO+V2G was examined through some changes in the load values, system parameters, and PV power generated. Comparisons between the suggested controller and both conventional integrators and (I+ECO) have been investigated.

It has been noted that the proposed (I+ECO+V2G) control scheme gathers the advantages of both the optimal and conventional controllers, besides the simple structure of ECO that is easy to implement. In addition, both MATLAB Simulink and RTS results have demonstrated the efficiency of the suggested (I+ECO+V2G) control technique. It was clear that the power system performance in the case of using the proposed (I+ECO+V2G) controller is more robust in the face of the load and parameters variations and PV fluctuations. In addition, more desirable performance can be achieved using the proposed control scheme compared to the conventional integral control method. Furthermore, the digital results have proved that both (I+ECO) and (I+ECO+V2G) schemes can provide robust responses against load change and parameters uncertainties; but the suggested (I+ECO+V2G) can give better desirable and smooth responses. The future research direction can be presented as follows:

1. Evaluating the impact of integrating additional controllable loads, such as heat pumps, on the system.
2. Investigation of other optimal controllers such as linear quadratic gaussian.
3. Comparing with other recent control methods.
4. Application of a new optimization technique on the addressed system.

## Abbreviations

∆*Pgi*: The output deviation of the governor
∆*Pmi*: The output deviation of the turbine
∆*fi*: The frequency change
∆*PLi*: The load variation
∆*Pci*: Supplementary control action
Hi: constant of equivalent inertia
Di: Coefficient of equivalent damping
Ri: Characteristic of speed droop
*Tti*,*Tgi*: Time constants of turbine and governor
ACEi: The area control error
Bi: The frequency bias factor
Tij: Coefficient of tie-line
∆*Ptie*: *i* The whole change of tie-line power among area *i* and the connected areas
∆*vi*: The interface of the control area, $v_{i}=\sum_{j=1,j\neq i}^{N}T_{ij}\Delta f_{j}$

## References

[1] IEA, “Transport biofuels–Renewables 2022 –Analysis,” IEA. 2022. [Online]. Available: https://www.iea.org/reports/renewables-2022/transport-biofuels

[2] Mahmoud M. M., Khalid Ratib M., Aly M. M., and Abdel-Rahim A. M. M., “Wind-driven permanent magnet synchronous generators connected to a power grid: Existing perspective and future aspects,” *Wind Eng*., vol. 46, no. 1, pp. 189–199, 2022, doi: 10.1177/0309524X211022728

[3] Awad M., Mahmoud M. M., Elbarbary Z. M. S., Mohamed Ali L., Fahmy S. N., and Omar A. I., “Design and analysis of photovoltaic/wind operations at MPPT for hydrogen production using a PEM electrolyzer: Towards innovations in green technology,” *PLoS One*, vol. 18, no. 7, p. e0287772, 2023, doi: 10.1371/journal.pone.0287772 37471326 PMC10358930

[4] Zhang X., Wang Y., Yuan X., Shen Y., Lu Z., and Wang Z., “Adaptive Dynamic Surface Control with Disturbance Observers for Battery/Supercapacitor-based Hybrid Energy Sources in Electric Vehicles,” *IEEE Trans*. *Transp*. *Electrif*., 2022, doi: 10.1109/TTE.2022.3194034

[5] Daraz A., Malik S. A., Azar A. T., Aslam S., Alkhalifah T., and Alturise F., “Optimized Fractional Order Integral-Tilt Derivative Controller for Frequency Regulation of Interconnected Diverse Renewable Energy Resources,” *IEEE Access*, vol. 10, pp. 43514–43527, 2022, doi: 10.1109/ACCESS.2022.3167811

[6] Elmetwaly A. H. et al., “Modeling, Simulation, and Experimental Validation of a Novel MPPT for Hybrid Renewable Sources Integrated with UPQC: An Application of Jellyfish Search Optimizer,” *Sustainability*, vol. 15, no. 6, p. 5209, 2023, doi: 10.3390/su15065209

[7] Ibrahim N. F., Alkuhayli A., Beroual A., Khaled U., and Mahmoud M. M., “Enhancing the Functionality of a Grid-Connected Photovoltaic System in a Distant Egyptian Region Using an Optimized Dynamic Voltage Restorer : Application of Artificial Rabbits Optimization,” 2023. 10.3390/s23167146 PMC10458465 37631683

[8] Ewais A. M., Elnoby A. M., Mohamed T. H., Mahmoud M. M., Qudaih Y., and Hassan A. M., “Adaptive frequency control in smart microgrid using controlled loads supported by real-time implementation,” *PLoS One*, vol. 18, no. 4, p. e0283561, 2023, doi: 10.1371/journal.pone.0283561 37043463 PMC10096276

[9] Singh K., Dahiya M., Grover A., Adlakha R., and Amir M., “An effective cascade control strategy for frequency regulation of renewable energy based hybrid power system with energy storage system,” *J*. *Energy Storage*, vol. 68, 2023, doi: 10.1016/j.est.2023.107804

[10] Dahiya P., Mukhija P., Saxena A. R., and Arya Y., “Comparative performance investigation of optimal controller for AGC of electric power generating systems,” *Automatika*, vol. 57, no. 4, pp. 902–921, 2016, doi: 10.7305/automatika.2017.12.1707

[11] Singh K. and Arya Y., “Tidal turbine support in microgrid frequency regulation through novel cascade Fuzzy-FOPID droop in de-loaded region,” *ISA Trans*., vol. 133, pp. 218–232, 2023, doi: 10.1016/j.isatra.2022.07.010 35879113

[12] Daraz A., Malik S. A., Basit A., Aslam S., and Zhang G., “Modified FOPID Controller for Frequency Regulation of a Hybrid Interconnected System of Conventional and Renewable Energy Sources,” *Fractal Fract*., vol. 7, no. 1, 2023, doi: 10.3390/fractalfract7010089

[13] Mahmoud M. M. et al., “Evaluation and Comparison of Different Methods for Improving Fault Ride-Through Capability in Grid-Tied Permanent Magnet Synchronous Wind Generators,” *Int*. *Trans*. *Electr*. *Energy Syst*., vol. 2023, 2023, [Online]. Available: https://downloads.hindawi.com/journals/itees/2023/7717070.pdf

[14] Singh K., Amir M., and Arya Y., “Optimal dynamic frequency regulation of renewable energy based hybrid power system utilizing a novel TDF-TIDF controller,” *Energy Sources*, *Part A Recover*. *Util*. *Environ*. *Eff*., vol. 44, no. 4, pp. 10733–10754, 2022, doi: 10.1080/15567036.2022.2158251

[15] Wu F., Liu R., Xie Y., and Lyu J., “A modified power decoupling control strategy for a grid-connected inverter with a low switching frequency under unbalanced grid voltages,” *Energy Reports*, vol. 8, pp. 757–768, 2022, doi: 10.1016/j.egyr.2022.02.247

[16] Zhang G., Daraz A., Khan I. A., Basit A., Khan M. I., and Ullah M., “Driver Training Based Optimized Fractional Order PI-PDF Controller for Frequency Stabilization of Diverse Hybrid Power System,” *Fractal Fract*., vol. 7, no. 4, 2023, doi: 10.3390/fractalfract7040315

[17] Daraz A. et al., “Automatic generation control of multi-source interconnected power system using FOI-TD controller,” *Energies*, vol. 14, no. 18, 2021, doi: 10.3390/en14185867 PMC7678963 33216787

[18] Zhang S. and Leung K. C., “Joint Optimal Power Flow Routing and Vehicle-to-Grid Scheduling: Theory and Algorithms,” *IEEE Trans*. *Intell*. *Transp*. *Syst*., vol. 23, no. 1, pp. 499–512, 2022, doi: 10.1109/TITS.2020.3012489

[19] Kamel O. M., Diab A. A. Z., Mahmoud M. M., Al-Sumaiti A. S., and Sultan H. M., “Performance Enhancement of an Islanded Microgrid with the Support of Electrical Vehicle and STATCOM Systems,” *Energies*, vol. 16, no. 4, 2023, doi: 10.3390/en16041577

[20] Gay D., Rogers T., and Shirley R., “Small island developing states and their suitability for electric vehicles and vehicle-to-grid services,” *Util*. *Policy*, vol. 55, pp. 69–78, 2018, doi: 10.1016/j.jup.2018.09.006

[21] Das S. and Panda S., “An optimized fractional order cascade controller for frequency regulation of power system with renewable energies and electric vehicles,” *Energy Syst*., vol. 14, no. 1, pp. 171–195, 2023, doi: 10.1007/s12667-021-00461-9

[22] Mazumder M. and Debbarma S., “EV Charging Stations with a Provision of V2G and Voltage Support in a Distribution Network,” *IEEE Syst*. *J*., vol. 15, no. 1, pp. 662–671, 2021, doi: 10.1109/JSYST.2020.3002769

[23] Arya Y., “Effect of electric vehicles on load frequency control in interconnected thermal and hydrothermal power systems utilising cf-foidf controller,” *IET Gener*. *Transm*. *Distrib*., vol. 14, no. 14, pp. 2666–2675, 2020, doi: 10.1049/iet-gtd.2019.1217

[24] Mosaad M. I. and Salem F., “LFC based adaptive PID controller using ANN and ANFIS techniques,” *J*. *Electr*. *Syst*. *Inf*. *Technol*., vol. 1, no. 3, pp. 212–222, 2014, doi: 10.1016/j.jesit.2014.12.004

[25] Mohamed S. A., Anwer N., and Mahmoud M. M., “Solving optimal power flow problem for IEEE-30 bus system using a developed particle swarm optimization method: towards fuel cost minimization,” *Int*. *J*. *Model*. *Simul*., vol. 00, no. 00, pp. 1–14, 2023, doi: 10.1080/02286203.2023.2201043

[26] Boudjemai H. et al., “Application of a Novel Synergetic Control for Optimal Power Extraction of a Small-Scale Wind Generation System with Variable Loads and Wind Speeds,” *Symmetry (Basel)*., vol. 15, no. 2, 2023.

[27] Ramadan H. S., Padmanaban S., and Mosaad M. I., “Metaheuristic-based Near-Optimal Fractional Order PI Controller for On-grid Fuel Cell Dynamic Performance Enhancement,” *Electr*. *Power Syst*. *Res*., vol. 208, 2022, doi: 10.1016/j.epsr.2022.107897

[28] Çelik E., Öztürk N., and Arya Y., “Advancement of the search process of salp swarm algorithm for global optimization problems,” *Expert Syst*. *Appl*., vol. 182, 2021, doi: 10.1016/j.eswa.2021.115292

[29] Sharma G., Krishnan N., Arya Y., and Panwar A., “Impact of ultracapacitor and redox flow battery with JAYA optimization for frequency stabilization in linked photovoltaic-thermal system,” *Int*. *Trans*. *Electr*. *Energy Syst*., vol. 31, no. 5, 2021, doi: 10.1002/2050-7038.12883

[30] Metwally Mahmoud M., “Improved current control loops in wind side converter with the support of wild horse optimizer for enhancing the dynamic performance of PMSG-based wind generation system,” *Int*. *J*. *Model*. *Simul*., 2022, doi: 10.1080/02286203.2022.2139128

[31] Alhejji A. and Mosaad M. I., “Performance enhancement of grid-connected PV systems using adaptive reference PI controller,” *Ain Shams Eng*. *J*., vol. 12, no. 1, pp. 541–554, 2021, doi: 10.1016/j.asej.2020.08.006

[32] Mosaad M. I., Alenany A., and Abu-Siada A., “Enhancing the performance of wind energy conversion systems using unified power flow controller,” *IET Gener*. *Transm*. *Distrib*., vol. 14, no. 10, pp. 1922–1929, 2020, doi: 10.1049/iet-gtd.2019.1112

[33] Mahmoud M. M. et al., “Application of Whale Optimization Algorithm Based FOPI Controllers for STATCOM and UPQC to Mitigate Harmonics and Voltage Instability in Modern Distribution Power Grids,” *Axioms*, vol. 12, no. 5, 2023, doi: 10.3390/axioms12050420

[34] Rajesh K. S. and Dash S. S., “Load frequency control of autonomous power system using adaptive fuzzy based PID controller optimized on improved sine cosine algorithm,” *J*. *Ambient Intell*. *Humaniz*. *Comput*., vol. 10, no. 6, pp. 2361–2373, 2019, doi: 10.1007/s12652-018-0834-z

[35] Xia S., Bu S. Q., Luo X., Chan K. W., and Lu X., “An Autonomous Real-Time Charging Strategy for Plug-In Electric Vehicles to Regulate Frequency of Distribution System with Fluctuating Wind Generation,” *IEEE Trans*. *Sustain*. *Energy*, vol. 9, no. 2, pp. 511–524, 2018, doi: 10.1109/TSTE.2017.2746097

[36] Das S., Acharjee P., and Bhattacharya A., “Charging Scheduling of Electric Vehicle Incorporating Grid-to-Vehicle and Vehicle-to-Grid Technology Considering in Smart Grid,” *IEEE Trans*. *Ind*. *Appl*., vol. 57, no. 2, pp. 1688–1702, 2021, doi: 10.1109/TIA.2020.3041808

[37] Mohamed T. H., Shabib G., and Ali H., “Distributed load frequency control in an interconnected power system using ecological technique and coefficient diagram method,” *Int*. *J*. *Electr*. *Power Energy Syst*., vol. 82, pp. 496–507, 2016, doi: 10.1016/j.ijepes.2016.04.023

[38] Tassadduq S. S. et al., “Ecological Distribution Patterns of Wild Grasses and Abiotic Factors,” *Sustain*., vol. 14, no. 18, 2022, doi: 10.3390/su141811117

[39] Yedavalli R. K. and Devarakonda N., “Ecological sign stability and its use in robust control design for aerospace applications,” in *Proceedings of the IEEE International Conference on Control Applications*, 2008, pp. 912–917. doi: 10.1109/CCA.2008.4629683

[40] Wang H. H., Finney M. A., Song Z. L., Wang Z. S., and Li X. C., “Ecological techniques for wildfire mitigation: Two distinct fuelbreak approaches and their fusion,” *Forest Ecology and Management*, vol. 495. 2021. doi: 10.1016/j.foreco.2021.119376

[41] Joint Committee For Guides In Metrology, “Evaluation of measurement data—Guide to the expression of uncertainty in measurement,” 2008. [Online]. Available: http://www.bipm.org/en/publications/guides/gum.html

[42] Joint Committee for Guides in Metrology, “Evaluation of measurement data—Supplement 1 to the ‘Guide to the expression of uncertainty in measurement’—Propagation of distributions using a Monte Carlo method,” *Evaluation*, vol. JCGM 101:2, p. 90, 2008.

[43] Yedavalli R. K., *Robust control of uncertain dynamic systems*: *A linear state space approach*. 2014. doi: 10.1007/978-1-4614-9132-3

[44] Yedavalli R. K. and Devarakonda N., “Sign-stability concept of ecology for control design with aerospace applications,” *J*. *Guid*. *Control*. *Dyn*., vol. 33, no. 2, pp. 333–346, 2010, doi: 10.2514/1.46196

[45] kai Feng Z., jing Niu W., and tian Cheng C., “Optimization of hydropower reservoirs operation balancing generation benefit and ecological requirement with parallel multi-objective genetic algorithm,” *Energy*, vol. 153, pp. 706–718, 2018, doi: 10.1016/j.energy.2018.04.075

[46] Mohamed T. H., Zaki Diab A. A., and Hussein M. M., “Application of linear quadratic Gaussian and coefficient diagram techniques to distributed load frequency control of power systems,” *Appl*. *Sci*., vol. 5, no. 4, pp. 1603–1615, 2015, doi: 10.3390/app5041603

[47] I. S. 1559–2019, “1159–2019—IEEE Recommended Practice for Monitoring Electric Power Quality | IEEE Standard | IEEE Xplore,” *IEEE Std 1159–2019*, pp. 1–98, 2019.

